# ATHENA: an independently validated autophagy-related epigenetic prognostic prediction model of head and neck squamous cell carcinoma

**DOI:** 10.1186/s13148-023-01501-0

**Published:** 2023-06-09

**Authors:** Ziang Xu, Xinlei Chen, Xiaomeng Song, Xinxin Kong, Jiajin Chen, Yunjie Song, Maojie Xue, Lin Qiu, Mingzhu Geng, Changyue Xue, Wei Zhang, Ruyang Zhang

**Affiliations:** 1 Jiangsu Key Laboratory of Oral Diseases, Nanjing Medical University, 136 Hanzhong Road, Nanjing, 210029 Jiangsu China; 2 Department of Oral Special Consultation, Affiliated Stomatological Hospital of Nanjing Medical University, Nanjing, Jiangsu China; 3 Department of Oral and Maxillofacial Surgery, Affiliated Hospital of Stomatology, Nanjing Medical University, Nanjing, Jiangsu China; 4 Department of Biostatistics, Center for Global Health, School of Public Health, Nanjing Medical University, SPH Building Room 406, 101 Longmian Avenue, Nanjing, 211166 Jiangsu China; 5 Department of Implant Dentistry, Affiliated Stomatological Hospital of Nanjing Medical University, Nanjing, Jiangsu China; 6 China International Cooperation Center for Environment and Human Health, Nanjing Medical University, Nanjing, China; 7 Changzhou Medical Center, Nanjing Medical University, Changzhou, 213164 Jiangsu China

**Keywords:** Head and neck squamous cell carcinoma, DNA methylation, Gene–gene interaction, Autophagy, Prognostic prediction, Immune landscape

## Abstract

The majority of these existing prognostic models of head and neck squamous cell carcinoma (HNSCC) have unsatisfactory prediction accuracy since they solely utilize demographic and clinical information. Leveraged by autophagy-related epigenetic biomarkers, we aim to develop a better prognostic prediction model of HNSCC incorporating CpG probes with either main effects or gene–gene interactions. Based on DNA methylation data from three independent cohorts, we applied a 3-D analysis strategy to develop An independently validated auTophagy-related epigenetic prognostic prediction model of HEad and Neck squamous cell carcinomA (ATHENA). Compared to prediction models with only demographic and clinical information, ATHENA has substantially improved discriminative ability, prediction accuracy and more clinical net benefits, and shows robustness in different subpopulations, as well as external populations. Besides, epigenetic score of ATHENA is significantly associated with tumor immune microenvironment, tumor-infiltrating immune cell abundances, immune checkpoints, somatic mutation and immunity-related drugs. Taken together these results, ATHENA has the demonstrated feasibility and utility of predicting HNSCC survival (http://bigdata.njmu.edu.cn/ATHENA/).

**Supplementary Information:**

The online version contains supplementary material available at 10.1186/s13148-023-01501-0.

## Introduction

Head and neck squamous cell carcinoma (HNSCC) is an aggressive malignancy that includes a wide range of phenotypes such as cancers of the lip, oral cavity, larynx, nasopharynx, oropharynx and hypopharynx, which results in nearly 900,000 new cases and 450,000 deaths globally in year 2020 [1]. Despite recent breakthroughs in surgery, radiotherapy, chemotherapy, targeted therapy, and immunotherapy, the prognosis of HNSCC is still poor and the 5-year survival rate of HNSCC stagnates at about 50% [2]. In the past decades, great efforts have been made to carry out genetic [3], epigenetic [4], transcriptomic [5] and proteomic [6] studies of HNSCC survival, since effective biomarkers possess the capability to predict prognosis of the disease, and can help to diagnose disease in its early stage, which is essential to improve the overall survival of HNSCC. Therefore, a significant amount of HNSCC associated molecular biomarkers has emerged [7].

Autophagy is the collective term covering a number of catabolic pathways that regulate cellular homeostasis via lysosomal degradation and recycling of cytoplasmic components [8]. The role of autophagy in regulating cancer progression is complex and contradictory; its specific function depends on the cancer type and tumor stage [9, 10]. Autophagy could negatively or positively regulate cancer immunotherapy by degrading immune checkpoint protein, releasing pro-inflammatory cytokines, and generating or degradating antigens [11]. Change of autophagic flux is associated with cancer cell proliferation and metastasis [12], tumor stem cell phenotype [10], tumor malignancy [13], and lymph node metastasis [14] in HNSCC. During HNSCC treatment, regulation of autophagy may modulate cisplatin resistance [15, 16], and help overcome radiotherapy resistance [17].

DNA methylation is a heritable, reversible and one of the most fundamental epigenetic modifications, which regulates gene transcription [18]. Aberrant DNA methylation is also involved in the progression of cancer [19], and tracking the aberrant methylation contributes substantially to the prognostic prediction of cancer survival [20]. However, the effect of DNA methylation of autophagy-related genes (ARGs) on HNSCC survival requires further investigation. Furthermore, almost all prognostic models of HNSCC merely focus on predictors with main effects, but overlook predictors exhibiting gene–gene (G × G) interactions, which may also contribute to discovery of therapeutic targets and boost prognostic prediction accuracy [21, 22].

Hence, we performed a two-step designed study to develop An independently validated auTophagy-related prognostic prediction model of HEad and Neck squamous cell carcinomA (ATHENA) by incorporating epigenetic biomarkers with main effects and G × G interactions using all available data in The Cancer Genome Atlas (TCGA) and Gene Expression Omnibus (GEO), and also analyzed the relationships between the epigenetic predictors and immune landscape.

## Methods

### Study populations with DNA methylation data

The level-3 TCGA-HNSCC DNA methylation and clinical data is obtained from the UCSC XENA browser, whose tumor sites are mostly tongue, larynx or overlapped lesions of lip, oral cavity and pharynx. Two additional independent datasets with clinical and DNA methylation information are downloaded from GEO (GSE75537 [23] and GSE52793 [24]). GSE75537 includes tumor samples from 53 tongue squamous cell carcinomas, while GSE52793 is consisted of oral rinse samples from 82 oral squamous cell carcinoma patients.

### Quality control process for DNA methylation data

DNA methylation is assessed by the Illumina Infinium Human Methylation 450 Array. We use R package *CHAMP* to process level-3 data from TCGA and GEO. Ineligible CpG probes are removed if they met any of the quality control (QC) criteria: (i) non-CpG probes, (ii) common single nucleotide polymorphisms (SNPs) located in the position of the CpG probe or 10 bp flanking regions, (iii) cross-reactive probes, (iv) sex chromosome probes, (v) deletion rates > 20%, (vi) failed QC in either TCGA or GEO cohorts. Type I and II probe correction is processed using Beta-Mixture Quantile (BMIQ) normalization. Additional file 1: Figure S1 describes the details of the QC process. Subjects without overall survival time are also removed. Finally, 634 subjects (Table 1) and 361,065 CpG probes are remained in the subsequent association analysis.

**Table 1:** Demographic and clinical characteristics of subjects in different datasets

Biomarker screening	Discovery phase	Validation phase		Combined dataset
Model development	Training set	Internal testing set	External testing set	
Characteristics	TCGA	GSE75537	GSE52793	
Number of samples	499	53	82	634
Age at diagnosis (years)	61.08 ± 11.92	49.36 ± 13.47	–	–
Age at diagnosis, *N* (%)				
< 40	18 (3.6)	16 (30.2)	–	34 (6.2)
40–49	58 (11.6)	15 (28.3)	–	73 (13.2)
50–59	144 (28.9)	7 (13.2)	–	151 (27.4)
≥ 60	279 (55.9)	15 (28.3)	–	294 (53.2)
Unknown	0	0	82	82
Gender, *N* (%)				
Male	366 (73.3)	42 (79.3)	–	408 (73.9)
Female	133 (26.7)	11 (20.7)	–	144 (26.1)
Unknown	0	0	82	82
T stage, *N* (%)				
I	33 (6.8)	13 (24.5)	–	46 (8.6)
II	142 (29.3)	15 (28.3)	–	157 (29.2)
III	130 (26.9)	12 (22.7)	–	142 (26.4)
IV	179 (37.0)	13 (24.5)	–	192 (35.8)
Unknown	15	0	82	97
N stage, *N* (%)				
0	238 (49.9)	25 (47.2)	–	263 (49.6)
1	80 (16.8)	8 (15.1)	–	88 (16.6)
2	152 (31.9)	20 (37.7)	–	172 (32.5)
3	7 (1.4)	0 (0)	–	7 (1.3)
Unknown	22	0	82	104
M stage, *N* (%)				
0	469 (99.0)	45 (100.0)	–	514 (99.0)
1	5 (1.0)	0 (0)	–	5 (1.0)
Unknown	25	8	82	115
Smoking status, *N* (%)				
Yes	378 (77.3)	–	–	378 (77.3)
No	111 (22.7)	–	–	111 (22.7)
Unknown	10	53	82	145
Race, *N* (%)				
White	426 (87.8)	–	–	426 (87.8)
Black or African American	47 (9.7)	–	–	47 (9.7)
Asian	10 (2.1)	–	–	10 (2.1)
American Indian or Alaska Native	2 (0.4)	–	–	2 (0.4)
Unknown	14	53	82	149

### Study populations with gene expression data and somatic mutation data

In the TCGA cohort, 499 HNSCC patients have complete mRNA sequencing data and 493 patients have complete somatic mutation data. TCGA mRNA sequencing data processing and quality control are performed by the TCGA working group. Level-3 mRNA expression data that downloaded from the UCSC XENA database is composed of fragments per kilobase per million mapped reads (FPKM) values, and is transformed into transcripts per kilobase million (TPM) values for association analysis. The expression value of each gene is also transformed on a log_2_ scale before association analysis.

### Quality control process for gene expression and somatic mutation data

After quality control, 44 HNSCC subjects with missing overall survival time or clinical information are excluded, yielding a total of 455 HNSCC subjects with complete mRNA sequencing data and 449 subjects with complete somatic mutation data for subsequent association analysis.

### Definition of autophagy-related biomarkers

We focus on a total of 232 ARGs defined by the Human Autophagy Database (HADb, http://www.autophagy.lu/), which is an online database that stores a complete set of human encoded genes related to autophagy as described in the published literature. After QC, there are 4306 CpG probes for association analysis.

### Statistical analysis

#### Model development and validation of ATHENA

**Fig. 1:**
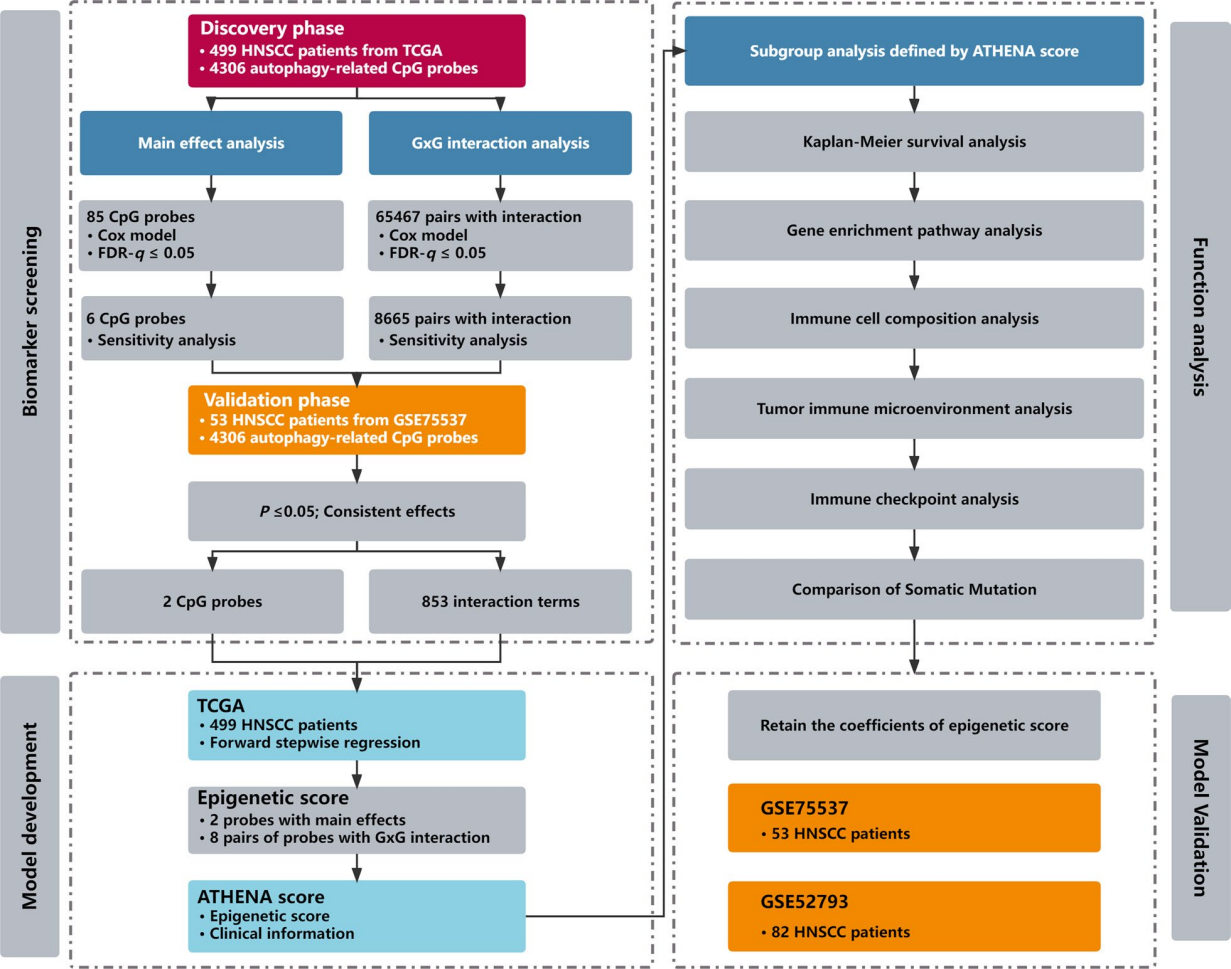
Flow chart of study design and statistical analyses

We depicted the study design and workflow in Fig. 1. For the development and validation of the ATHENA, we applied a 3-D strategy which was originally proposed in our previous study [21], including Double Types of Effects, Double Steps of Screening, and Double Steps of Modeling.

1. *Double Types of Effects* We aimed to include epigenetic predictors with either main effects or G × G interactions. (i) To test the first type of effect (main effect), we utilized Cox proportional hazards model adjusted for covariates including age, gender, smoking status and TNM stage. (ii) To test the second type of effect (G × G interaction), we again used Cox proportional hazards model adjusted for covariates aforementioned.
2. *Double Steps of Screening* We adopted a two-step design to scan biomarkers with either significant main effects or G × G interactions on HNSCC overall survival. (i) In the step of biomarker screening, we tested those two types of effects through Cox models aforementioned in TCGA as a discovery phase. Multiple test corrections were performed by controlling the false discovery rate (FDR) at the 5% level. To reduce the impact of outliers, we deleted methylation values out of range mean ± 3 × standard deviation (SD), and retested these effects as a sensitivity analysis. Hazard ratios (HR) and 95% confidence intervals (CIs) were calculated for incremental methylation per 1% level. (ii) In the step of biomarker validation, we confirmed their significances in GSE75537 as a validation phase. Significant biomarkers were finally retained if they met all following criteria: (i) FDR-*q* ≤ 0.05 in the discovery phase; (ii) *P* ≤ 0.005 in the sensitivity analysis of discovery phase; (iii) *P* ≤ 0.05 in the validation phase; (iv) consistent direction of effects across two phases.
3. *Double Steps of Modeling* (i) In the step of model development, we applied forward stepwise regression strategy to select the final predictors for ATHENA from significant biomarkers survived from *Double Steps of Screening* in TCGA data as a training set. Biomarkers retained in the multivariable Cox model were identified by the likelihood ratio test with *P*_entry_ ≤ 0.05 and *P*_removal_ > 0.05. (ii) In the step of model validation for ATHENA, model performance was assessed in one internal testing set (GSE75537) and another external testing set (GSE52793) with coefficients of all epigenetic predictors the same as those trained in TCGA.

Kaplan–Meier survival curves are drawn to illustrate the survival difference among patients with different risk groups. The accuracy of prediction is represented using the time-dependent receiver operating characteristic (ROC) curve, and is measured by the area under the ROC curve (AUC), which can be obtained from R package *timeROC*. The 95% CI and *P* value of AUC improvement are calculated by 1000-time bootstrap resampling. The concordance index (*C*-index), an average accuracy of predictive survival across all follow-up years, is also utilized to estimate predictive performance. Furthermore, we performed decision curve analysis (DCA) to evaluate clinical benefits by using ATHENA to screen out these patients at high risk of death. Stratification analysis of ATHENA scores is displayed within subgroups stratified by age, gender, smoking status, TNM stage and occurrence site using the R package *forestplot*. Finally, one nomogram is generated with R package *regplot*. To facilitate application of ATHENA model, we release an online calculator (http://bigdata.njmu.edu.cn/ATHENA/), which can immediately return predicted survival rates and 95% CIs at any time point between 0 and 120 months when inputting values of predictors for a HNSCC patient, based on an interactive web-based Kaplan–Meier survival curve.

#### Immune landscape analysis of epigenetic predictors of ATHENA

Potential genes *trans*-regulated by epigenetic predictors of ATHENA are identified by genome-wide correlation analysis using linear regression model and Cox model adjusted for the same covariates aforementioned in TCGA cohort. Functional annotation and gene enrichment pathway analysis based on Kyoto Encyclopedia of Genes and Genomes (KEGG) and Gene Ontology (GO) for potential *trans*-regulated genes are performed using R Package *WebGestaltR*. The ESTIMATE algorithm is used based on gene expression data to explore the pattern of tumor immune microenvironment (TIME) among subgroups [25], and *CIBERSORT*, a linear support vector regression-based deconvolution algorithm [26], is performed to determine the composition of 22 tumor-infiltrating immune cells (TIICs). We explored the difference of immune checkpoint expression in epigenetic score subgroups, and also performed the correlation analysis between immune checkpoints expression and epigenetic score of ATHENA. Then, based on the somatic mutation data from TCGA, we conducted a differential analysis of genomic mutations between high- and low-risk groups using R package *maftools*. Finally, we explored immunity-related drugs targeting epigenetic predictors using the DrugBank database (https://go.drugbank.com/) [27].

Continuous variables are summarized as mean ± SD, while categorized variables are described by frequency (*n*) and proportion (%) in description analysis. All statistical analyses are performed in R software (version 4.0.3, The R Foundation for Statistical Computing, Vienna, Austria), unless otherwise specified.

## Results

### ATHENA model development

First, 85 CpG probes with main effects and 65,467 pairs of CpG probes with G × G interactions were identified (FDR-*q* ≤ 0.05) to be possibly associated with overall survival in the discovery phase. Of them, 6 probes with main effects and 8,665 pairs of probes with G × G interactions passed the sensitivity analysis. Finally, 2 probes and 853 pairs of probes were confirmed with robust significance in the validation phase, and were defined to be candidate epigenetic predictors. By using forward stepwise regression strategy in TCGA cohort as training set, we constructed a Cox model including 2 CpG probes with main effects (Additional file 1: Table S1) and 8 pairs of CpG probes with G × G interactions (Additional file 1: Table S2), which were used to calculate the epigenetic score (Additional file 1: Table S3). The ATHENA score was defined as a weighted linear combination of epigenetic score and clinical score (Additional file 1: Tables S4, S5), where weights were coefficients derived from Cox model.

### ATHENA model evaluation and validation

**Fig. 2:**
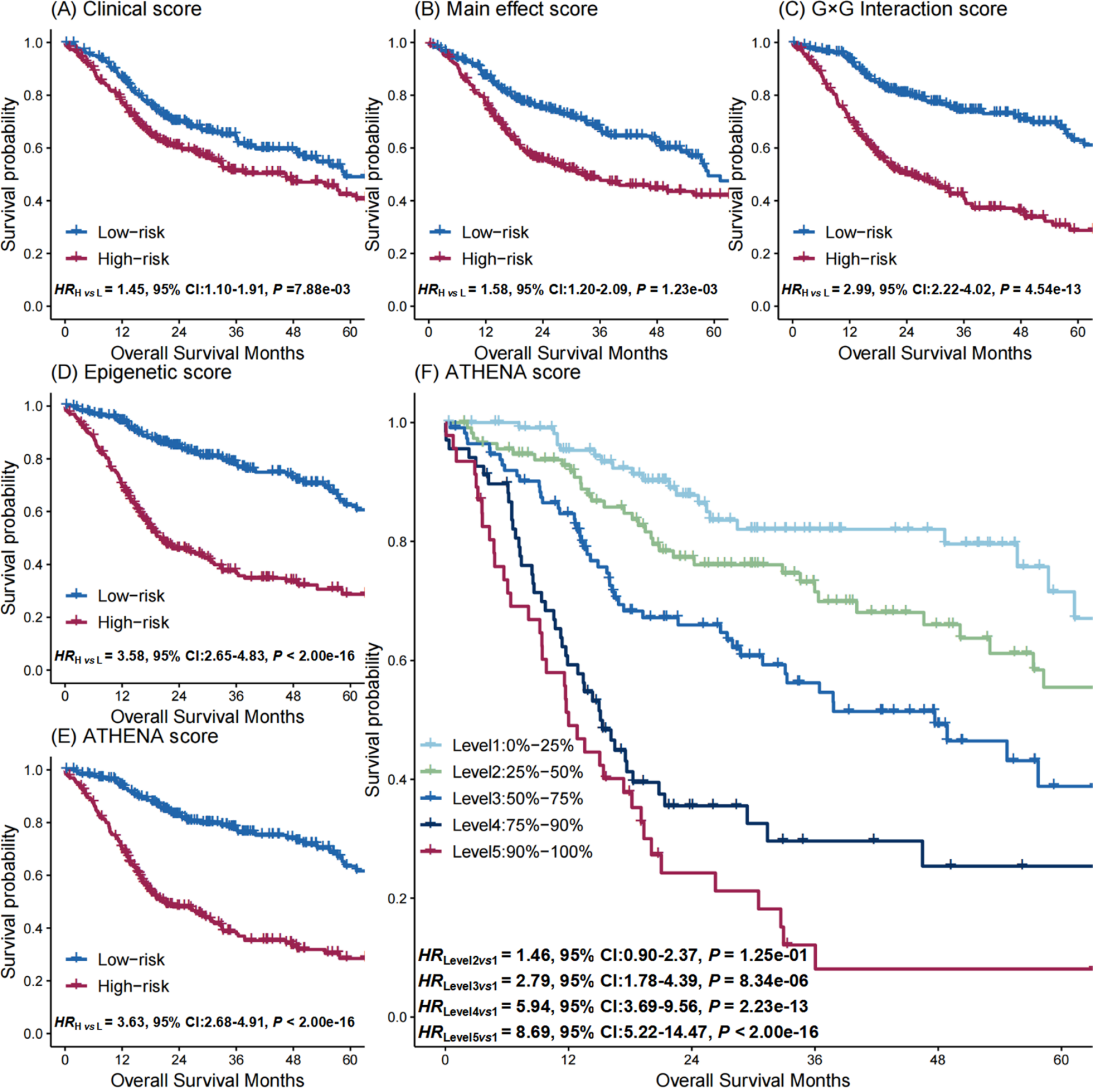
Kaplan–Meier survival curves for high- and low-risk HNSCC patients. The high- and low risk groups are defined by the median of **A** clinical score, **B** main effect score, **C** G × G interaction score, **D** epigenetic score, and **E** ATHENA score. **F** Discriminative ability of the ATHENA score is illustrated by Kaplan–Meier survival curves of six groups, defined by quantiles at 20%, 40%, 60%, 80% and 90% of ATHENA score

To evaluate the discriminative ability of biomarkers of ATHENA, patients were categorized into low- and high-risk groups based on the median of (i) clinical score which was a weighted linear combination of demographic and clinical factors; (ii) main effect score which was a weighted linear combination of epigenetic biomarkers with significant main effects; (iii) G × G interaction score which was a weighted linear combination of epigenetic biomarkers with significant G × G interactions; (iv) epigenetic score which was a a weighted linear combination of main effect score and G × G interaction score; and (v) ATHENA score which was a weighted linear combination of clinical and epigenetic scores, respectively. Compared to the low-risk group, the high-risk group was associated with worse survival in TCGA cohort, exhibiting a gradually increasing hazard ratio (HR) (HR_Clinical score_ = 1.45, 95% CI 1.10–1.91, *P* = 7.88 × 10^–03^; HR_Main effect score_ = 1.58, 95% CI 1.20–2.09, *P* = 1.23 × 10^–03^; HR_G×G Interaction score_ = 2.99, 95% CI 2.22–4.02, *P* = 4.54 × 10^–13^; HR_Epigenetic score_ = 3.58, 95% CI 2.65–4.83, *P* < 2.00 × 10^–16^; HR_ATHENA score_ = 3.63, 95% CI 2.68–4.91, *P* < 2.00 × 10^–16^) (Fig. 2A–E). To further illustrate the discriminative ability of the ATHENA score, we categorized patients by the quintiles and the 90 percentile of the score in the TCGA cohort. We observed a dose–response association between higher-percentile groups and higher mortality risk (HR_Level 5 *vs* 1_ = 8.69, 95% CI 5.22–14.47, *P* < 2.00 × 10^–16^; HR_Level 4 *vs* 1_ = 5.94, 95% CI 3.69–9.56, *P* = 2.23 × 10^–13^; HR_Level 3 *vs* 1_ = 2.79, 95% CI 1.78–4.39, *P* = 8.34 × 10^–06^; HR_Level 2 *vs* 1_ = 1.46, 95% CI 0.90–2.37, *P* = 1.25 × 10^–01^) (Fig. 2F).

**Fig. 3:**
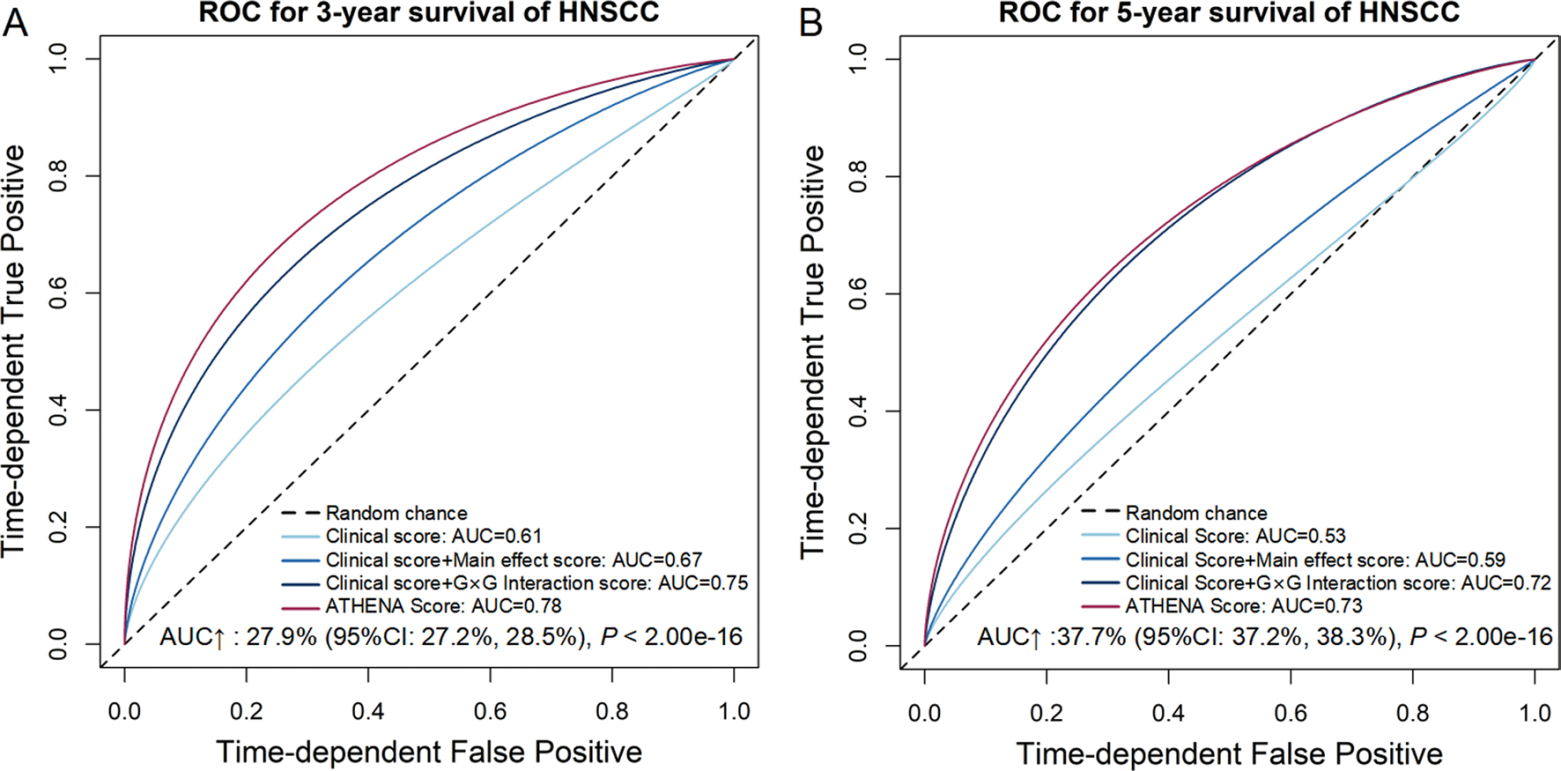
ROC curves of different prognostic prediction models using different combinations of clinical information, epigenetic predictors with main effects and G × G interactions. ROC curves are presented for both **A** 36-month and **B** 60-month survival prediction. The AUC increase (%) is evaluated by comparing ATHENA model and the model with only covariates. *P* values and 95% CIs are calculated by using 1000 bootstrap samples

**Fig. 4:**
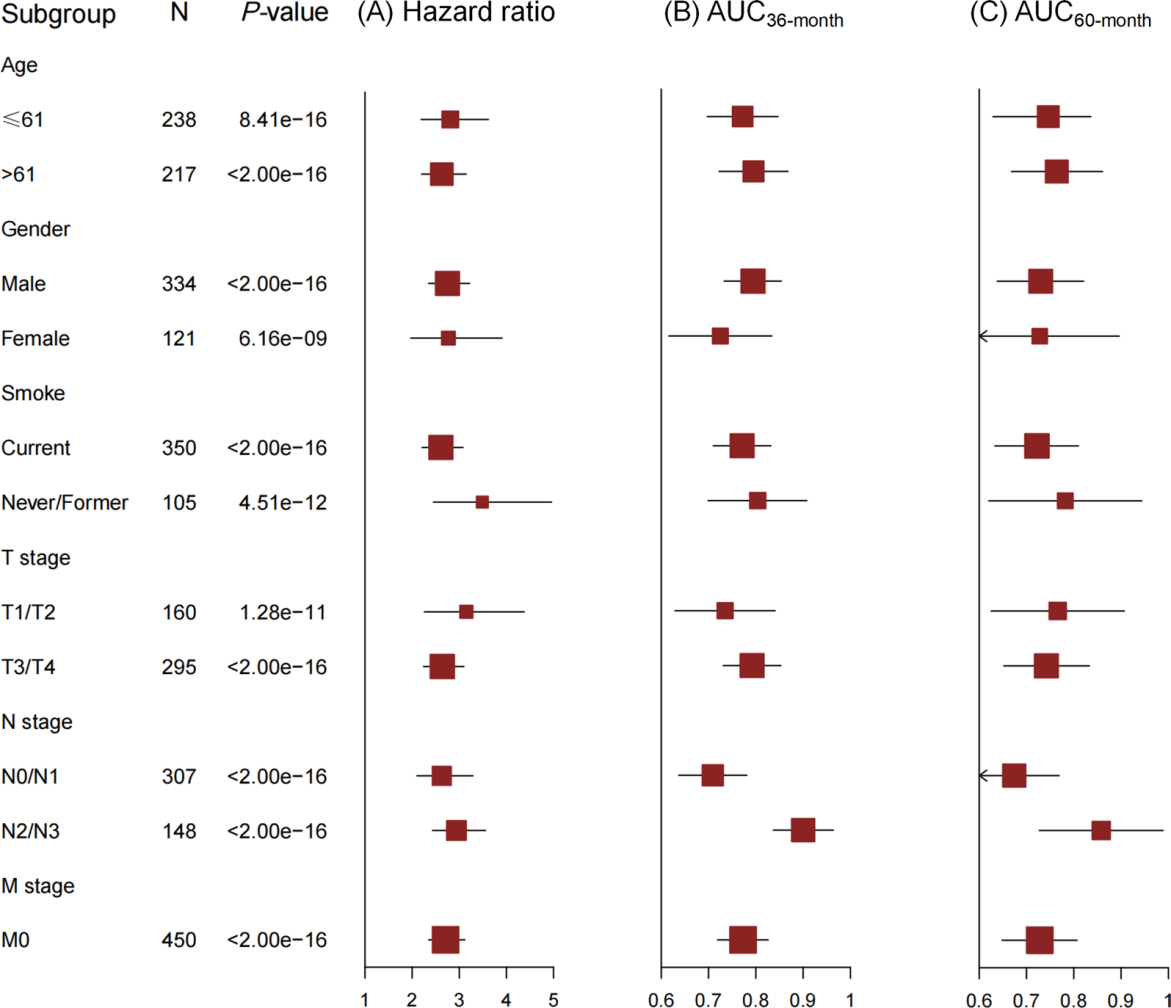
Subgroup analyses of ATHENA score. **A** Hazard ratio is used to evaluate the association between ATHENA score and HNSCC survival. The AUC is used to measure the prediction accuracy of ATHENA for **B** 36-month and **C** 60-month survival

**Fig. 5:**
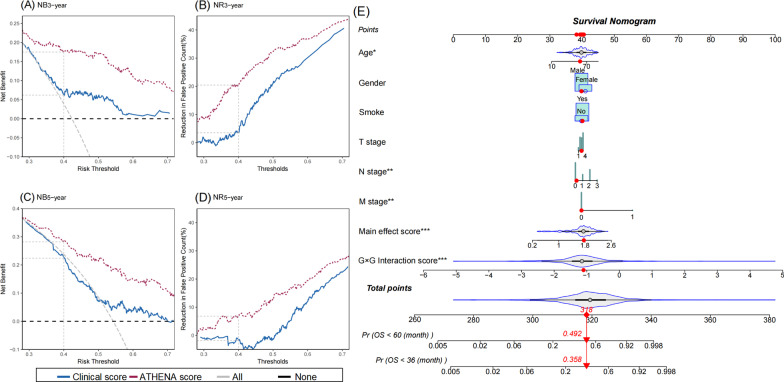
Decision curve analysis and nomogram of ATHENA. The net benefit (NB) and net reduction (NR) of patients avoided unnecessary interventions are given at threshold (0.4) for both 36-month (**A**, **B**) and 60-month (**C**, **D**) survival. **E** For the nomogram of ATHENA model, the value of each predictor can be converted into the corresponding points according to the axis in the top of nomogram. The sum of points for each predictor can correspond to the total points axis at the bottom of the nomogram and further be used to estimate the patient's 36- and 60-month survival rate

We then evaluated the prediction performance of these biomarkers. The model with only clinical score had a limited prediction ability (AUC_36-month_ = 0.61, AUC_60-month_ = 0.53, *C*-index = 0.60), and the prediction accuracy increased by adding main effect score (AUC_36-month_ = 0.67, AUC_60-month_ = 0.59, *C*-index = 0.64) or G × G interaction score (AUC_36-month_ = 0.75, AUC_60-month_ = 0.72, *C*-index = 0.72). Further, by adding epigenetic score, the AUC increased by 27.9% (95% CI 27.2–28.5%, *P* < 2.00 × 10^–16^) and 37.7% (95% CI 37.2–38.3%, *P* < 2.00 × 10^–16^) for 3-year and 5-year survival prediction, respectively (Fig. 3A, B). And, ATHENA achieved an acceptable prediction accuracy (AUC_36-month_ = 0.78, AUC_60-month_ = 0.73, *C*-index = 0.73). In further subgroup analyses in subpopulations stratified by age, gender, smoking status and TNM stage, ATHENA still presented robust discriminative ability with HR ranging from 2.61 (95% CI 2.21–3.08, *P* < 2.00 × 10^–16^) to 3.49 (95% CI 2.45–4.96, *P* = 4.51 × 10^–12^), and exhibited reasonable prediction accuracy with AUC ranging from 0.71 (95% CI 0.64–0.78) to 0.90 (95% CI 0.84–0.97) for 36-month survival prediction, and 0.67 (95% CI 0.58–0.77) to 0.86 (95% CI 0.73–0.99) for 60-month survival prediction, respectively (Fig. 4A–C). Considering the potential tissue heterogeneity, we also evaluated ATHENA model in different subgroups by occurrence sites, and observed its robust performance (Additional file 1: Figure S2A–C). DCA showed that ATHENA presented more clinical net benefits than model with only clinical and demographic indicators (Fig. 5A–D). To facilitate application of ATHENA in clinical practice, we developed a nomogram, which estimated patients’ 36- or 60-month survival (Fig. 5E).

Finally, for ATHENA model validation, we retained the coefficients of CpG probes when applying to two extra datasets (GSE75537 as an internal validation and GSE52793 as an external validation). ATHENA showed a satisfactory prediction accuracy in GSE75537 (AUC_36-month_ = 0.82, AUC_60-month_ = 0.80, *C*-index = 0.75) (Additional file 1: Figure S3A), while epigenetic score was still an independent and significant risk factor for prognosis (HR = 1.55, 95% CI 1.16–2.07, *P* = 3.09 × 10^–03^) (Additional file 1: Figure S3B). The AUC of ATHENA model in GSE52793 were limited because of the absence of covariates (AUC_36-month_ = 0.59, AUC_60-month_ = 0.62, *C*-index = 0.59) (Additional file 1: Figure S4A), while epigenetic score was again significantly associated with HNSCC overall survival (*P*_*p*=0, *q*=1_ = 2.39 × 10^–02^ and *P*_*p*=1, *q*=1_ = 2.97 × 10^–02^) as shown by Kaplan–Meier survival curves (Additional file 1: Figure S4B), which was confirmed by Harrington–Fleming test that was designed for the late or delayed effect of variable during the follow-up [28].

### *Trans*-regulation analyses of epigenetic predictors of ATHENA and immune landscape analysis

**Fig. 6:**
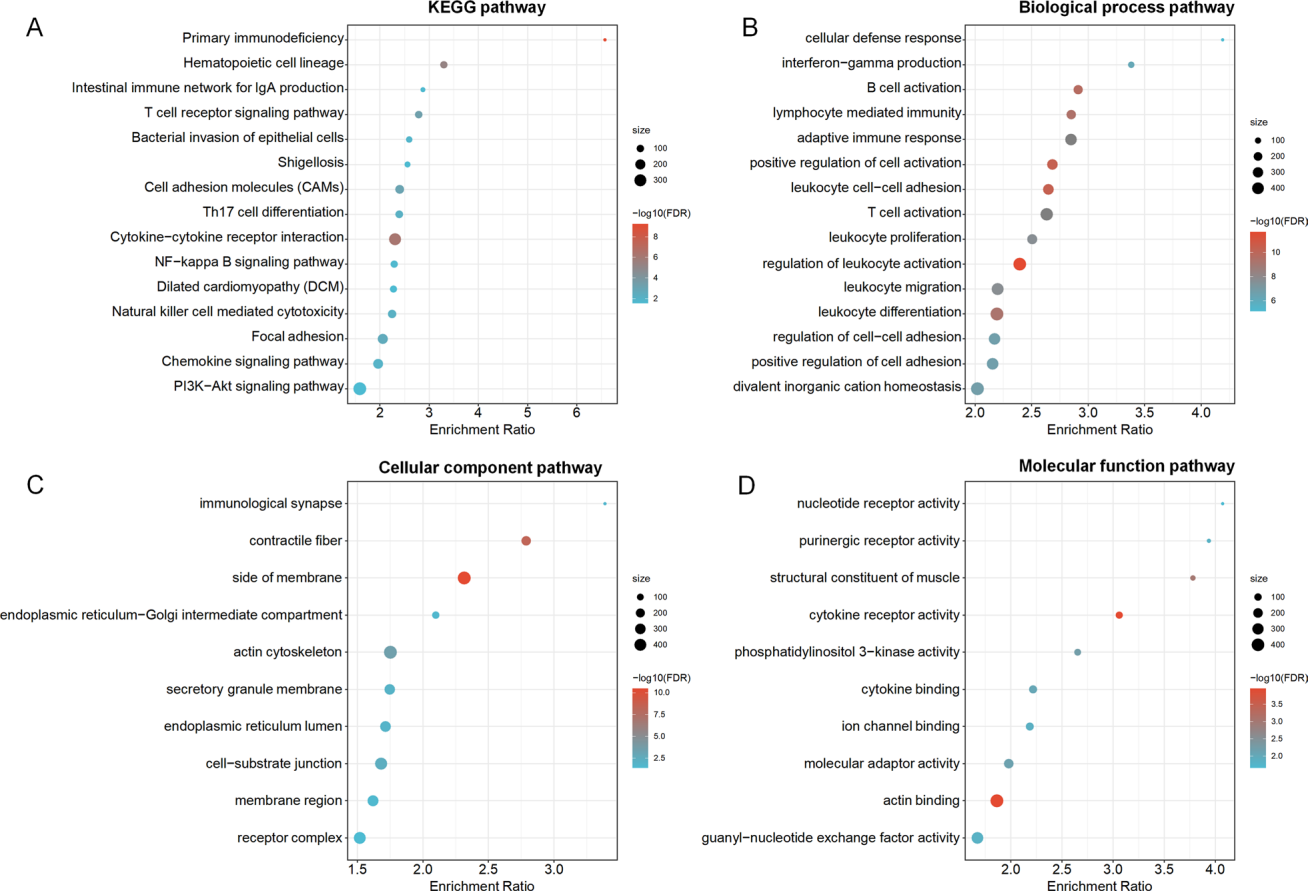
Significant pathways with genes *trans*-regulated by epigenetic predictors of ATHENA in gene enrichment pathway analysis. **A** The top 15 significant KEGG pathways, **B** the top 15 significant biological process pathways, **C** the top 10 significant cellular component pathways, and **D** the top 10 significant molecular function pathways were sorted by enrichment ratio

Genome-wide *trans-*regulation analyses by the linear regression model indicated that expressions of 6507 genes were significantly *trans*-regulated by the epigenetic predictors of ATHENA (FDR-*q* ≤ 0.05). Among them, 1564 genes were further significantly associated with HNSCC overall survival (*P* ≤ 0.05), which were evaluated by the Cox proportional hazards model adjusted for the same covariates aforementioned. KEGG enrichment analysis categorized gene probes into 21 pathways, including classic autophagy-related pathways such as *PI3K-Akt signaling pathway*, and GO annotation identified 65 biological process pathways, 10 cellular component pathways and 14 molecular function pathways, suggesting potential biological functions (Fig. 6A–D). By extracting CpG probes of autophagy-related genes in *PI3K-Akt signaling pathway*, and testing these biomarkers using the same criteria aforementioned, again, we observed 35 pairs of CpG probes with significant G × G interactions in discovery and validation phases, which could be potential drug target therapeutics to overcome autophagy in HNSCC (Additional file 1: Table S6).

**Fig. 7:**
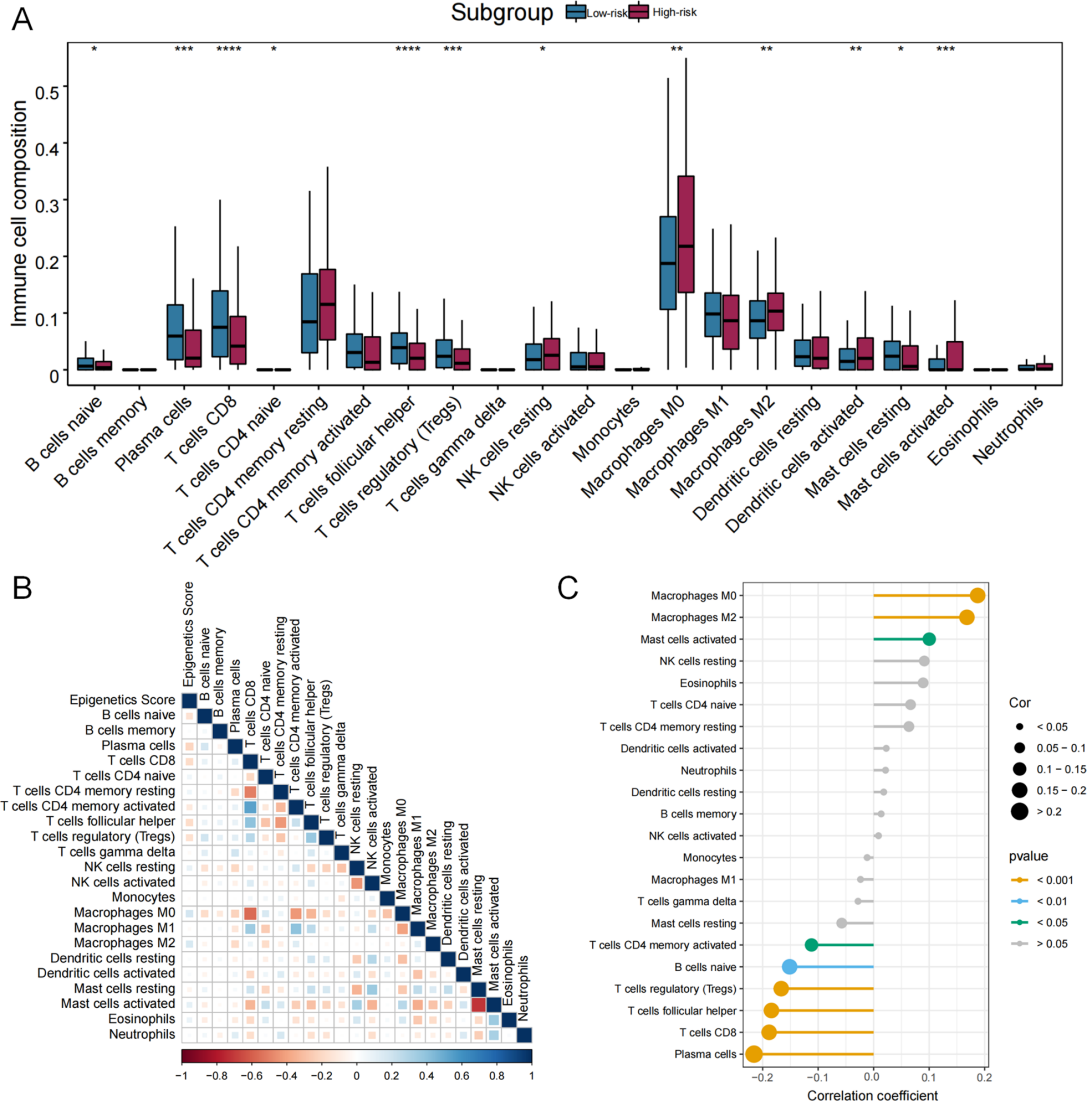
The association analysis between immune cells and epigenetic score of ATHENA. **A** The abundances of 22 immune cells are compared between high- and low- risk-groups. * means *P* < 0.05, ** means *P* < 0.01, *** means *P* < 0.001 and **** means *P* < 0.0001. **B** The correlation coefficients between immune cells and epigenetic score of ATHENA are derived from Pearson correlation analyses and are presented in a heatmap. **C** The correlation coefficients between immune cells and epigenetic score of ATHENA are derived from Pearson correlation analyses and these pairs are listed in lollipop chart

**Fig. 8:**
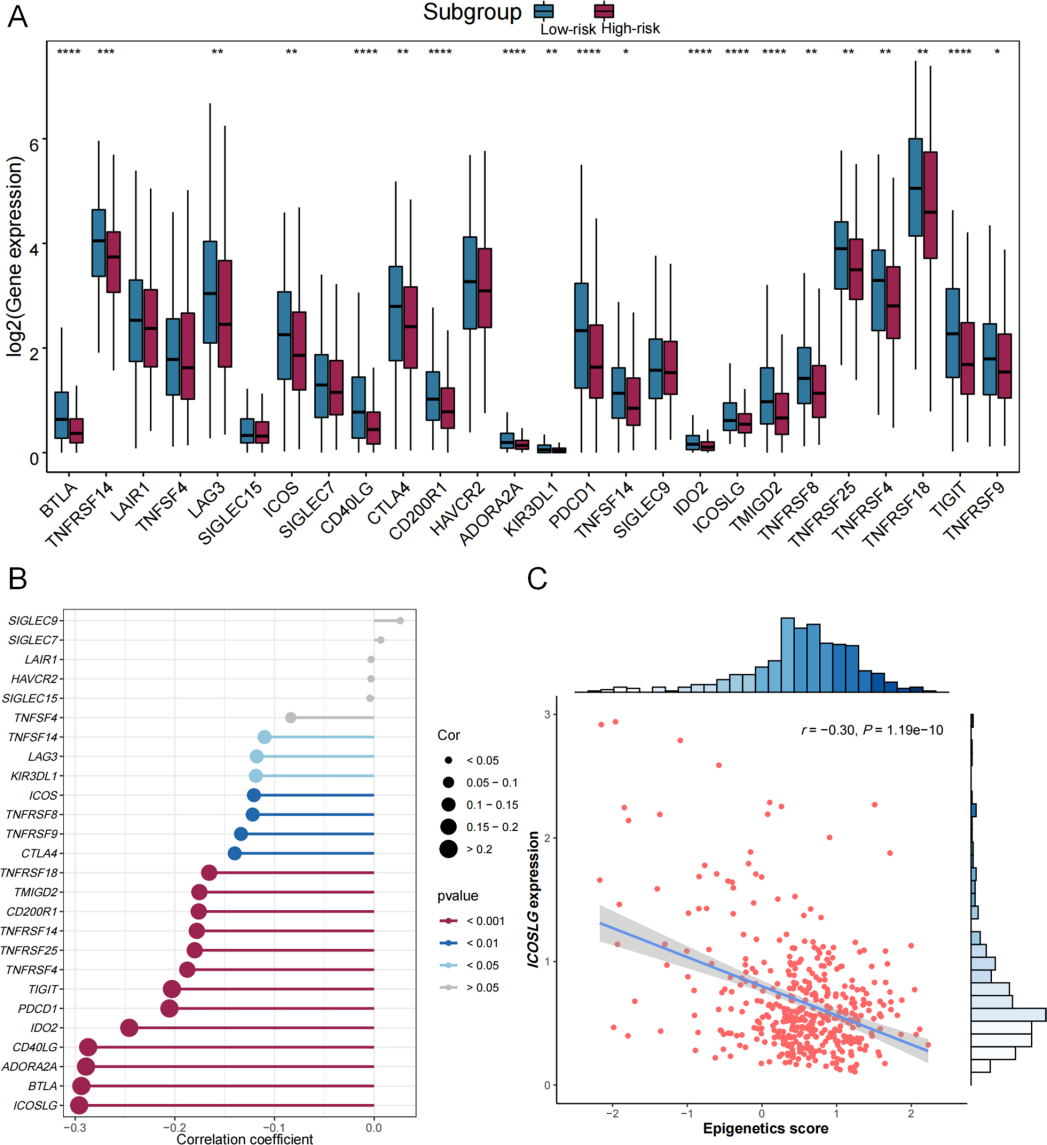
The association analysis between immune checkpoints and epigenetic score of ATHENA. **A** The gene expressions of 26 immune checkpoints are compared between high- and low-risk-groups. * means *P* < 0.05, ** means *P* < 0.01, *** means *P* < 0.001 and **** means *P* < 0.0001. **B** The correlation coefficients between immune checkpoints and epigenetic score of ATHENA are derived from Pearson correlation analyses and these pairs are listed in lollipop chart. **C** The scatter plot and linear regression analysis between epigenetic score of ATHENA and expression of *ICOSLG* with the strongest association

**Fig. 9:**
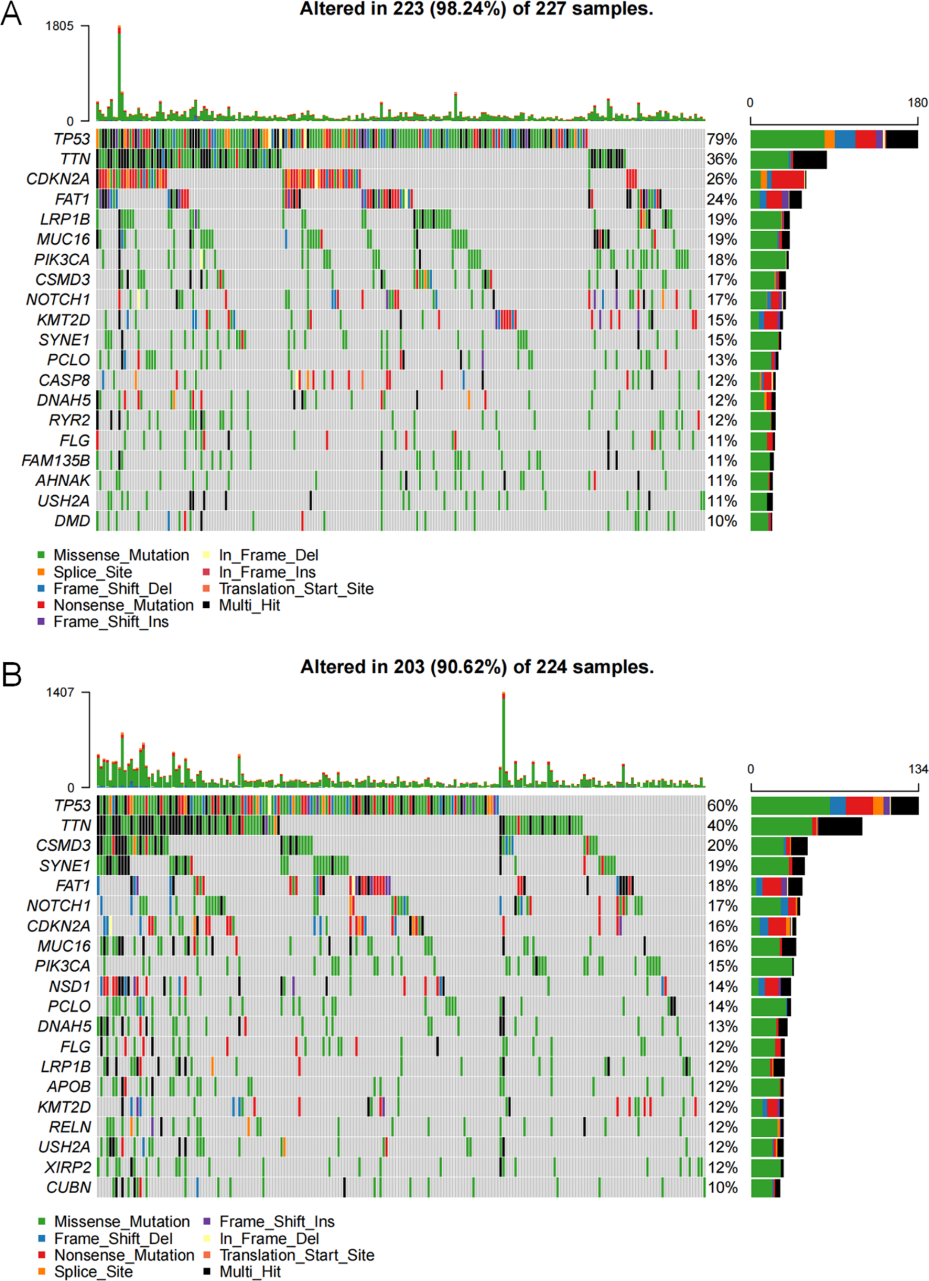
Waterfall plots of the top 20 somatic mutated genes in high- and low-risk groups. **A** The high-risk and **B** the low-risk group are defined by the median of the epigenetic score of ATHENA

Moreover, the compositions of 12 types of immune cells were differently distributed between low- and high-risk groups determined by median of epigenetic score of ATHENA (Fig. 7A), the correlation of epigenetic score of ATHENA with each immune cell composition varied a lot (Fig. 7B), including positive (e.g., with M0 Macrophages) and negative correlations (e.g., with Plasma cells) (Fig. 7C). Further, epigenetic score of ATHENA had statistically significant but very weak positive correlation with the stromal score (*r* = 0.11, *P* = 1.99 × 10^–02^), and showed no significant negative correlation with the immune score (*r* = − 0.09, *P* = 5.24 × 10^–02^) (Additional file 1: Figure S5). As a result, we checked the connectivity and correlations between epigenetic score of ATHENA and immune checkpoint genes. Almost all of the immune checkpoints genes were lower expressed in the high-risk group (Fig. 8A), thus suggested a negative correlation between immune checkpoint gene expressions and epigenetic score of ATHENA (Fig. 8B), especially *ICOSLG* (*r* = − 0.30, *P* = 1.19 × 10^–10^) (Fig. 8C). Then we used waterfall maps to investigate the differences in genomic mutations between the high- and low-risk groups. *TP53* (79%), *TTN* (36%), *CDKN2A* (26%), *FAT1* (24%) and *LRP1B* (19%) were the top 5 genes with the highest mutation frequencies in the high-risk group, while *TP53* (60%), *TTN* (40%), *CSMD3* (20%), *SYNE1* (19%) and *FAT1* (18%) were the top 5 genes in the low-risk group (Fig. 9A, B). Finally, numerous immunity-related drugs targeting genes, which epigenetic predictors located, have been documented (Additional file 1: Table S7), and, thereby ATHENA model may have potential roles in guiding immunotherapy.

## Discussion

One major reason of limited accuracy of the prognostic model is the solely use of demographic and clinical information. These unsatisfactory models cannot accurately indicate high-risk patients who require close follow-up and postoperative adjuvant therapy [29]. Hence, there is an urgent need to develop accurate prognostic prediction models aiding in clinical decisions [30]. Using available public HNSCC epigenetic data from three independent cohorts, we employed a 3-D analysis strategy to screen biomarkers and established an autophagy-related epigenetic prognostic prediction model, ATHENA. ATHENA showed acceptable prediction accuracy in both training and internal testing sets, and also showed fair accuracy and discrimination in external testing set with oral rinse samples of HNSCC patients, suggesting the robustness and clinical significance of ATHENA.

Gene–environment and gene–gene interactions provided additional insights into the biological mechanisms of complex diseases [31–33]. In our previous study [34], we explored epigenome-wide gene–age interaction and significantly improved the accuracy of prognostic prediction model of oral squamous cell carcinoma. ATHENA is the first attempt of an autophagy-related epigenetic prognostic model in HNSCC patients, and also one of the earliest explorations of G × G interaction of HNSCC overall survival on epigenome-wide scale. Our results showed that biomarkers with G × G interaction significantly improved the prediction accuracy of prognostic model of HNSCC and demonstrated the importance of complex association patterns among multiple factors in the study of complex diseases (e.g., HNSCC) again.

Interestingly, CpG probes located on *ITPR1* appeared several times in the interaction terms of ATHENA model. Inositol 1, 4, 5-trisphosphate receptor type 1 (*ITPR1*), located on chromosome 3, is a pivotal gene for autophagy [35, 36]. Expression of *ITPR1* can be upregulated by *EGOT* via RNA–RNA and RNA–protein interactions to enhance autophagy [37], meanwhile, showed association with tumorigenesis of cells squamous [38] and prognosis of HNSCC patients [39]. We hypothesize that *ITPR1* may be a hub gene of epigenome-wide, and even transcriptome-wide interaction of autophagy-related genes in HNSCC. In the enrichment pathway analysis, *trans-*regulated genes were significantly enriched in autophagy-related pathways or processes (*PI3K-Akt signaling pathway*; *NF-kappa B signaling pathway*), and classic processes of the extracellular matrix (ECM) (*Focal adhesion*). This suggests that there may also be a cooperative exchange between ECM and autophagy during progression of tumor in HNSCC patients, which is proved in other kinds of neoplasms [40, 41]. Notably, in our study, epigenetic score of ATHENA has statistically significant positive correlation with stromal scores, indicating that higher stromal scores may lead to poorer HNSCC survival and suggesting that higher ECM stiffness may affect autophagy [42].

In addition, the relationship between autophagy and immunity has been widely reported. Autophagy is upregulated in many cancers, which may support the growth, survival and malignancy of neoplasm, may suppress activation of the innate immune response, and may suppress the adaptive immune response and contribute to tumor immune evasion [43], suggesting epigenetic score of ATHENA is negatively correlated with immune score. Also, autophagy may reduce the ability of T cells to kill tumor cells [44], inhibit antigen presentation [45], which explains the negative correlation between epigenetic score of ATHENA and helper follicular T cells. The correlation between the epigenetic score and immune cell composition could be also partially explained by the biological process pathways enriched in *trans*-regulated genes. For example, the negative correlation between CD8 T cells and epigenetic score may be caused by impaired T cell activation due to increased tumor malignancy. Moreover, inhibition of autophagy may enhance response of targeted therapy and blockade of immune checkpoints [46, 47]. In our study, there was also a clear inverse correlation between epigenetic score and immune checkpoint expression, which is also consistent with previous researches [48, 49], indicating that HNSCC is an immunosuppressive malignancy. Finally, genes involved in epigenetic score were transcriptional predictors with immune relevance, which can be immunotherapeutic targets.

Our study has several strengths. First, to our knowledge, this may be the first study to investigate G × G interaction on HNSCC survival on epigenome-wide scale, which provides new insights into the prognosis of HNSCC patients. Second, we adopt an effective 3-D strategy for biomarker screening and model construction, and focus on biomarkers with either significant main effects or G × G interactions, which can improve the accuracy of the prediction model. Third, we perform internal and external model validation of ATHENA, therefore, confirm the accuracy and extrapolation of ATHENA in HNSCC patients. Finally, we provide a web-based tool to facilitate the application of ATHENA.

We also acknowledge some limitations. First, the sample sizes of the discovery and validation cohorts are unbalanced, which may affect our results. Though we performed a comprehensive database search, only three available HNSCC DNA methylation datasets with overall survival information were suitable for our study, including TCGA (*n* = 499), GSE75537 (*n* = 53) and GSE52793 (*n* = 82). We performed strict correction of type I error and sensitivity analysis in TCGA cohort, and again validated significant signals in GSE75537 cohort to reduce the false positive probability. Indeed, limited sample size of GSE75537 yields to limited statistical power of confirming the significance of epigenetic predictors. Anyway, we still observed 2 CpG probes with significant main effects and 853 pairs of CpG probes with significant G × G interactions, which indicating our results are conservative. But, we envision more available database with large sample size and more identified epigenetic predictors in future, which will probably improve the accuracy of ATHENA. Second, sample origins vary across three different cohorts. HNSCC tumor samples in TCGA are composed of 23 types of tissues by occurrence site, including tongue, larynx, overlapped lesion of lip, oral cavity and pharynx, floor of mouth, etc. While, GSE75537 includes merely tongue tumor samples, and GSE52793 is consisted of oral rinse samples. Though sample heterogeneity exists among different types of tissues, under the strict premise of retaining the coefficients of epigenetic biomarkers instead of retraining the model, ATHENA still reflects acceptable prediction accuracy and discrimination ability in all three cohorts, indicating its robustness. Besides, ATHENA still retains statistical significance and prediction accuracy in almost all occurrence site subgroups with sufficient sample size (Additional file 1: Figure S2), which suggests well generalization ability of ATHENA model again. Finally, since the majority of samples in the TCGA cohort are Caucasians (87.8%), generalization of our results to the other ethnicities should be cautioned.

## Conclusion

We propose ATHENA, an accurate and independently validated prognostic prediction model of HNSCC incorporating autophagy-related epigenetic biomarkers with either main effects or G × G interactions. A free and user-friendly online tool is released at http://bigdata.njmu.edu.cn/ATHENA/.

## Web resources

TCGA: https://portal.gdc.cancer.gov

GEO: https://www.ncbi.nlm.nih.gov/geo/

UCSC Xena browser: https://xenabrowser.net

Human Autophagy Database (HADb): http://www.autophagy.lu/

The DrugBank database: https://go.drugbank.com/

## Supplementary Information

**Additional file 1.** Supplementary File.

## Data Availability

The datasets analyzed during the current study are available in the TCGA (https://portal.gdc.cancer.gov). The independent validation cohorts are available in the GEO (https://www.ncbi.nlm.nih.gov/geo/) (GSE75537 and GSE52793).

## Abbreviations

HNSCC: Head and neck squamous cell carcinoma
ARG: Autophagy-related gene
ATHENA: An independently validated auTophagy-related prognostic prediction model of HEad and Neck squamous cell carcinomA
TCGA: The Cancer Genome Atlas
GEO: Gene Expression Omnibus
QC: Quality control
SNP: Single nucleotide polymorphism
BMIQ: Beta-mixture quantile
FPKM: Fragments per kilobase per million mapped reads
TPM: Transcripts per kilobase million
HADb: The Human Autophagy Database
FDR: False discovery rate
SD: Standard deviation
HR: Hazard ratio
CI: Confidence interval
ROC: Receiver operating characteristic
AUC: Area under the receiver operating characteristic curve
*C*-index: Concordance index
DCA: Decision curve analysis
KEGG: Kyoto Encyclopedia of Genes and Genomes
GO: Gene Ontology
TIME: Tumor immune microenvironment
TIICs: Tumor-infiltrating immune cells
