# Supplementary material for: ATHENA: an independently validated autophagy-related epigenetic prognostic prediction model of head and neck squamous cell carcinoma
Source: Clin Epigenetics. 2023 Jun 9;15:97. doi: 10.1186/s13148-023-01501-0 (PMC10257287; doi:10.1186/s13148-023-01501-0)

Supplementary files

**Table S1**. Association results for two significant CpG probes with main effects derived from Cox regression models adjusted for covariates.

| CpG | Discovery phase | | | | | Validation phase | | | | Sensitivity analysis | | | |
| --- | --- | --- | --- | --- | --- | --- | --- | --- | --- | --- | --- | --- | --- |
|  | *HR* | 95% CI | | *P* | FDR*-q* | *HR* | 95% CI | | *P* | *HR* | 95% CI | | *P* |
| cg18368845 | 1.018 | 1.008 | 1.027 | 3.41×10^-04^ | 2.10×10^-02^ | 1.089 | 1.011 | 1.173 | 2.42×10^-02^ | 1.015 | 1.005 | 1.026 | 4.80×10^-03^ |
| cg03485669 | 1.020 | 1.009 | 1.032 | 5.90×10^-04^ | 3.34×10^-02^ | 1.070 | 1.011 | 1.131 | 1.83×10^-02^ | 1.021 | 1.010 | 1.033 | 3.07×10^-04^ |

**Table S2**. Association results for six pairs of CpG probes with significant G×G interactions derived from Cox regression models adjusted for covariates.

| Index | CpG1 | CpG2 | Discovery phase | | | | | Validation phase | | | | Sensitivity analysis | | | |
| --- | --- | --- | --- | --- | --- | --- | --- | --- | --- | --- | --- | --- | --- | --- | --- |
|  |  |  | *HR* | 95% CI | | *P* | FDR*-q* | *HR* | 95% CI | | *P* | *HR* | 95% CI | | *P* |
| 1 | cg11638200 | cg18576374 | 1.005 | 1.004 | 1.007 | 4.59×10^-12^ | 1.81×10^-07^ | 1.442 | 1.088 | 1.911 | 1.09×10^-02^ | 1.068 | 1.035 | 1.101 | 3.13×10^-05^ |
| 2 | cg04110105 | cg21024916 | 1.002 | 1.001 | 1.002 | 1.15×10^-08^ | 5.41×10^-05^ | 1.008 | 1.002 | 1.014 | 1.26×10^-02^ | 1.002 | 1.001 | 1.003 | 1.01×10^-03^ |
| 3 | cg03184452 | cg23662097 | 0.999 | 0.999 | 1.000 | 1.15×10^-05^ | 5.98×10^-03^ | 0.998 | 0.996 | 0.999 | 1.22×10^-02^ | 0.999 | 0.999 | 1.000 | 1.15×10^-05^ |
| 4 | cg00241355 | cg24531520 | 1.015 | 1.008 | 1.022 | 2.07×10^-05^ | 8.74×10^-03^ | 1.055 | 1.001 | 1.112 | 4.74×10^-02^ | 1.024 | 1.007 | 1.040 | 4.15×10^-03^ |
| 5 | cg14780845 | cg23662097 | 0.993 | 0.990 | 0.996 | 2.99×10^-05^ | 1.12×10^-02^ | 0.987 | 0.975 | 0.998 | 2.25×10^-02^ | 0.993 | 0.989 | 0.997 | 4.80×10^-04^ |
| 6 | cg05332270 | cg19671246 | 1.003 | 1.002 | 1.005 | 9.00×10^-05^ | 2.22×10^-02^ | 1.079 | 1.006 | 1.157 | 3.41×10^-02^ | 1.006 | 1.002 | 1.010 | 1.51×10^-03^ |
| 7 | cg10329001 | cg21722680 | 1.031 | 1.014 | 1.049 | 2.72×10^-04^ | 4.30×10^-02^ | 1.242 | 1.051 | 1.467 | 1.09×10^-02^ | 1.128 | 1.054 | 1.208 | 5.52×10^-04^ |
| 8 | cg14933257 | cg21382890 | 1.013 | 1.006 | 1.020 | 3.17×10^-04^ | 4.70×10^-02^ | 1.047 | 1.025 | 1.070 | 3.25×10^-05^ | 1.016 | 1.005 | 1.027 | 3.40×10^-03^ |

**Table S3**. Annotation information for CpG probes used to calculate epigenetic score of ATHENA, including 2 CpG probes with significant main effects and 8 pairs of CpG probes with significant G×G interactions screened out by forward stepwise regression if *P*_entry_ ≤ 0.05 and *P*_removal_ > 0.05.

| Effect type | Index | CpG probe | Gene | Chromosome | Region | CpG islands | Relation to CpG islands |
| --- | --- | --- | --- | --- | --- | --- | --- |
| Main effect | 1 | cg03485669 | *DLC1* | 8 | Body |  | Body-opensea |
|  | 2 | cg18368845 | *FOXO1* | 13 | Body |  | Body-opensea |
| Interaction | 1 | cg11638200 | *PRKCQ* | 10 | 5'UTR | chr10:6621907-6622642 | 5'UTR-island |
|  |  | cg18576374 | *RPTOR* | 17 | Body |  | Body-opensea |
|  | 2 | cg04110105 | *CCR2* | 3 | 5'UTR |  | 5'UTR-opensea |
|  |  | cg21024916 | *ITPR1* | 3 | 5'UTR | chr3:4534412-4535367 | 5'UTR-shore |
|  | 3 | cg03184452 | *DLC1* | 8 | Body |  | Body-opensea |
|  |  | cg23662097 | *ITPR1* | 3 | Body |  | Body-opensea |
|  | 4 | cg00241355 | *GAPDH* | 12 | TSS200 | chr12:6643261-6644607 | TSS200-island |
|  |  | cg24531520 | *RHEB* | 7 | TSS1500 | chr7:151216068-151217901 | TSS1500-shore |
|  | 5 | cg14780845 | *NBR1* | 17 | 5'UTR | chr17:41322972-41323363 | 5'UTR-island |
|  |  | cg23662097 | *ITPR1* | 3 | Body |  | Body-opensea |
|  | 6 | cg05332270 | *EIF2AK2* | 2 | TSS1500 | chr2:37383901-37384402 | TSS1500-island |
|  |  | cg19671246 | *PELP1* | 17 | TSS200 | chr17:4606984-4607722 | TSS200-island |
|  | 7 | cg10329001 | *SPNS1* | 16 | Body | chr16:28985881-28986661 | Body-island |
|  |  | cg21722680 | *TBK1* | 12 | 5'UTR | chr12:64845599-64846314 | 5'UTR-island |
|  | 8 | cg14933257 | *ATIC* | 2 | TSS200 | chr2:216176549-216177132 | TSS200-island |
|  |  | cg21382890 | *NFE2L2* | 2 | Body |  | Body-opensea |

# Table S4. Association results for CpG probes with main effects or G×G interactions derived from a multivariable Cox regression model in the TCGA training set.

| CpG probes | *HR* (95% CI) | *P* |
| --- | --- | --- |
| Main effect score |  |  |
| cg03485669 | 1.014 (1.002, 1.025) | 2.15×10^-02^ |
| cg18368845 | 1.015 (1.005, 1.025) | 3.07×10^-03^ |
|  |  |  |
| Epigenetic score |  |  |
| Main effect score | 2.737 (1.470, 5.097) | 1.51×10^-03^ |
| cg14933257 | 0.758 (0.599, 0.959) | 2.11×10^-02^ |
| cg21382890 | 0.945 (0.917, 0.973) | 1.60×10^-04^ |
| cg03184452 | 1.016 (1.002, 1.029) | 2.11×10^-02^ |
| cg23662097 | 1.064 (1.037, 1.092) | 2.05×10^-06^ |
| cg14780845 | 1.074 (1.036, 1.114) | 1.10×10^-04^ |
| cg04110105 | 0.984 (0.969, 1.000) | 4.66×10^-02^ |
| cg21024916 | 0.985 (0.973, 0.998) | 2.54×10^-02^ |
| cg00241355 | 0.820 (0.694, 0.970) | 2.02×10^-02^ |
| cg24531520 | 0.958 (0.925, 0.991) | 1.42×10^-02^ |
| cg05332270 | 0.988 (0.969, 1.007) | 1.97×10^-01^ |
| cg19671246 | 0.960 (0.922, 0.999) | 4.35×10^-02^ |
| cg10329001 | 0.957 (0.918, 0.998) | 3.96×10^-02^ |
| cg21722680 | 0.772 (0.605, 0.985) | 3.74×10^-02^ |
| cg11638200 | 0.996 (0.972, 1.019) | 7.13×10^-01^ |
| cg18576374 | 0.997 (0.978, 1.017) | 7.83×10^-01^ |
| cg14933257×cg21382890 | 1.016 (1.009, 1.024) | 1.73×10^-05^ |
| cg03184452×cg23662097 | 0.999 (0.999, 1.000) | 5.20×10^-04^ |
| cg23662097×cg14780845 | 0.992 (0.989, 0.996) | 2.34×10^-05^ |
| cg04110105×cg21024916 | 1.001 (1.000, 1.002) | 8.81×10^-04^ |
| cg00241355×cg24531520 | 1.015 (1.008, 1.022) | 2.18×10^-05^ |
| cg05332270×cg19671246 | 1.003 (1.001, 1.005) | 2.35×10^-04^ |
| cg10329001×cg21722680 | 1.023 (1.005, 1.041) | 1.06×10^-02^ |
| cg11638200×cg18576374 | 1.004 (1.003, 1.006) | 4.63×10^-08^ |

**Table S5**. Methods and formulas for calculating epigenetic score of ATHENA.

$$\text{ATHENA}_{\text{Main effect score}}=\sum_{i=1}^{L} \beta_{i}\times CpG_{i}$$

$$\text{ATHENA}_{\text{G}\times\text{G interaction score}}=\sum_{i=1}^{L} \beta_{i,1}\times CpG_{i,1}+\beta_{i,2}\times CpG_{i,2}+\beta_{i,3}\times CpG_{i,1}\times CpG_{i,2}$$

ATHENA_Main effect score_ = cg03485669×0.013495+cg18368845×0.015012

ATHENA_G×G interaction score_ =

-cg14933257×0.2767404-cg21382890×0.0570087+cg03184452×0.0155225+cg23662097×0.0621770+cg14780845×0.0715830-

cg04110105×0.0160793-cg21024916×0.0148114-cg00241355×0.1982574-cg24531520×0.0434670-cg05332270×0.0125744-

cg19671246×0.0411464-cg10329001×0.0438058-cg21722680×0.2586270-cg11638200×0.0044219-cg18576374×0.0026984+

cg14933257×cg21382890×0.0160678-cg03184452×cg23662097×0.0006354-cg23662097×cg14780845×0.0076471+

cg04110105×cg21024916×0.0010690+cg00241355×cg24531520×0.0149296+cg05332270×cg19671246×0.0029882+

cg10329001×cg21722680×0.0225824+cg11638200×cg18576374×0.0044100

ATHENA_Epigenetics score_ = ATHENA_Main effect score_×1.0067306+ATHENA_G×G interaction score_

# Table S6. Association results for 35 pairs of CpG probes mapping to autophagy-related genes in PI3K-Akt signaling pathway with significant G×G interactions derived from Cox regression models adjusted for covariates.

| id1 | id2 | TCGA | | | | | Sensitivity analysis | | | | GSE75537 | | | |
| --- | --- | --- | --- | --- | --- | --- | --- | --- | --- | --- | --- | --- | --- | --- |
|  |  | *HR* | 95% CI | | *P* | FDR-*q* | *HR* | 95% CI | | *P* | *HR* | 95% CI | | *P* |
| cg00611675 | cg03394237 | 1.111 | 1.064 | 1.159 | 1.38×10^-06^ | 1.67×10^-03^ | 1.118 | 1.043 | 1.199 | 1.68×10^-03^ | 1.223 | 1.084 | 1.380 | 1.08×10^-03^ |
| cg00611675 | cg07991600 | 1.028 | 1.018 | 1.038 | 3.48×10^-08^ | 1.24×10^-04^ | 1.040 | 1.017 | 1.064 | 6.57×10^-04^ | 1.343 | 1.04 | 1.734 | 2.40×10^-02^ |
| cg01052512 | cg01203549 | 1.049 | 1.024 | 1.075 | 1.17×10^-04^ | 2.91×10^-02^ | 1.046 | 1.020 | 1.073 | 5.82×10^-04^ | 1.141 | 1.007 | 1.293 | 3.89×10^-02^ |
| cg01500570 | cg25373579 | 1.004 | 1.002 | 1.006 | 7.21×10^-06^ | 5.06×10^-03^ | 1.004 | 1.003 | 1.006 | 3.09×10^-06^ | 1.027 | 1.000 | 1.054 | 4.72×10^-02^ |
| cg02045224 | cg07991600 | 1.033 | 1.016 | 1.051 | 1.31×10^-04^ | 3.12×10^-02^ | 1.063 | 1.024 | 1.104 | 1.56×10^-03^ | 1.639 | 1.135 | 2.368 | 8.45×10^-03^ |
| cg02198582 | cg25663524 | 1.003 | 1.002 | 1.004 | 6.16×10^-07^ | 9.58×10^-04^ | 1.003 | 1.001 | 1.004 | 1.11×10^-04^ | 1.017 | 1.005 | 1.029 | 4.69×10^-03^ |
| cg02910299 | cg07754880 | 1.007 | 1.004 | 1.011 | 8.76×10^-05^ | 2.48×10^-02^ | 1.014 | 1.007 | 1.022 | 8.00×10^-05^ | 1.044 | 1.011 | 1.078 | 9.42×10^-03^ |
| cg03394237 | cg08734093 | 1.152 | 1.088 | 1.221 | 1.60×10^-06^ | 1.84×10^-03^ | 1.229 | 1.068 | 1.414 | 3.90×10^-03^ | 2.102 | 1.447 | 3.053 | 9.61×10^-05^ |
| cg03394237 | cg23305046 | 1.144 | 1.078 | 1.214 | 9.72×10^-06^ | 6.21×10^-03^ | 1.205 | 1.066 | 1.362 | 2.95×10^-03^ | 1.324 | 1.034 | 1.695 | 2.59×10^-02^ |
| cg05121697 | cg12127162 | 1.026 | 1.016 | 1.036 | 2.88×10^-07^ | 5.74×10^-04^ | 1.033 | 1.013 | 1.053 | 8.90×10^-04^ | 1.034 | 1.013 | 1.055 | 1.15×10^-03^ |
| cg05231706 | cg21961852 | 1.001 | 1.001 | 1.002 | 8.09×10^-05^ | 2.36×10^-02^ | 1.004 | 1.002 | 1.007 | 5.27×10^-04^ | 1.035 | 1.005 | 1.066 | 2.13×10^-02^ |
| cg06096901 | cg07922924 | 1.002 | 1.001 | 1.002 | 5.22×10^-05^ | 1.83×10^-02^ | 1.001 | 1.001 | 1.002 | 5.45×10^-04^ | 1.005 | 1.001 | 1.009 | 8.56×10^-03^ |
| cg07530194 | cg11335969 | 1.004 | 1.002 | 1.007 | 9.30×10^-05^ | 2.56×10^-02^ | 1.006 | 1.003 | 1.009 | 6.43×10^-05^ | 1.028 | 1.000 | 1.056 | 4.90×10^-02^ |
| cg07655693 | cg12571687 | 1.068 | 1.034 | 1.104 | 8.81×10^-05^ | 2.49×10^-02^ | 1.166 | 1.050 | 1.294 | 3.93×10^-03^ | 1.336 | 1.031 | 1.731 | 2.85×10^-02^ |
| cg07922924 | cg12785535 | 1.004 | 1.002 | 1.006 | 2.25×10^-04^ | 4.27×10^-02^ | 1.008 | 1.003 | 1.014 | 3.57×10^-03^ | 1.038 | 1.015 | 1.063 | 1.30×10^-03^ |
| cg08128650 | cg24180621 | 1.002 | 1.001 | 1.003 | 6.51×10^-05^ | 2.11×10^-02^ | 1.003 | 1.001 | 1.006 | 1.63×10^-03^ | 1.012 | 1.003 | 1.021 | 6.88×10^-03^ |
| cg08248705 | cg11283404 | 1.009 | 1.005 | 1.013 | 7.64×10^-06^ | 5.28×10^-03^ | 1.013 | 1.005 | 1.022 | 1.73×10^-03^ | 1.131 | 1.046 | 1.222 | 1.94×10^-03^ |
| cg08595102 | cg19134705 | 1.306 | 1.173 | 1.455 | 1.14×10^-06^ | 1.47×10^-03^ | 1.405 | 1.156 | 1.708 | 6.21×10^-04^ | 1.192 | 1.006 | 1.413 | 4.27×10^-02^ |
| cg09326546 | cg14618923 | 1.098 | 1.046 | 1.152 | 1.48×10^-04^ | 3.36×10^-02^ | 1.107 | 1.032 | 1.188 | 4.54×10^-03^ | 1.469 | 1.206 | 1.789 | 1.32×10^-04^ |
| cg09420993 | cg25291404 | 1.002 | 1.001 | 1.002 | 2.80×10^-06^ | 2.64×10^-03^ | 1.002 | 1.001 | 1.003 | 3.21×10^-04^ | 1.011 | 1.004 | 1.019 | 3.52×10^-03^ |
| cg11283404 | cg27051224 | 1.027 | 1.013 | 1.041 | 2.01×10^-04^ | 4.01×10^-02^ | 1.026 | 1.008 | 1.044 | 4.30×10^-03^ | 1.055 | 1.015 | 1.096 | 6.94×10^-03^ |
| cg11335969 | cg11637695 | 1.005 | 1.003 | 1.006 | 1.49×10^-08^ | 6.90×10^-05^ | 1.004 | 1.001 | 1.007 | 2.56×10^-03^ | 1.039 | 1.003 | 1.077 | 3.59×10^-02^ |
| cg16780847 | cg27238079 | 1.016 | 1.007 | 1.024 | 1.79×10^-04^ | 3.74×10^-02^ | 1.014 | 1.006 | 1.023 | 4.82×10^-04^ | 1.037 | 1.007 | 1.068 | 1.38×10^-02^ |
| cg16782602 | cg27394817 | 1.123 | 1.057 | 1.194 | 1.88×10^-04^ | 3.84×10^-02^ | 1.107 | 1.040 | 1.179 | 1.50×10^-03^ | 2.782 | 1.359 | 5.698 | 5.15×10^-03^ |
| cg18198734 | cg21219851 | 1.001 | 1.001 | 1.002 | 1.48×10^-04^ | 3.36×10^-02^ | 1.004 | 1.001 | 1.006 | 1.23×10^-03^ | 1.011 | 1.004 | 1.018 | 1.16×10^-03^ |
| cg02240665 | cg15476425 | 0.949 | 0.925 | 0.974 | 7.07×10^-05^ | 2.19×10^-02^ | 0.953 | 0.927 | 0.981 | 1.07×10^-03^ | 0.685 | 0.586 | 0.801 | 2.05×10^-06^ |
| cg02851047 | cg20062492 | 0.996 | 0.995 | 0.998 | 1.30×10^-04^ | 3.09×10^-02^ | 0.997 | 0.995 | 0.998 | 2.69×10^-04^ | 0.968 | 0.944 | 0.992 | 9.76×10^-03^ |
| cg03046247 | cg04658243 | 0.998 | 0.997 | 0.999 | 2.95×10^-11^ | 7.75×10^-07^ | 0.997 | 0.995 | 0.999 | 3.69×10^-03^ | 0.987 | 0.978 | 0.996 | 4.53×10^-03^ |
| cg03354771 | cg06256295 | 0.961 | 0.941 | 0.980 | 1.13×10^-04^ | 2.85×10^-02^ | 0.955 | 0.931 | 0.980 | 3.74×10^-04^ | 0.934 | 0.877 | 0.994 | 3.21×10^-02^ |
| cg03354771 | cg06538549 | 0.984 | 0.976 | 0.992 | 1.17×10^-04^ | 2.91×10^-02^ | 0.984 | 0.974 | 0.994 | 1.97×10^-03^ | 0.971 | 0.944 | 0.998 | 3.89×10^-02^ |
| cg03923774 | cg16488565 | 0.927 | 0.892 | 0.964 | 1.24×10^-04^ | 3.02×10^-02^ | 0.933 | 0.896 | 0.970 | 5.25×10^-04^ | 0.808 | 0.696 | 0.939 | 5.29×10^-03^ |
| cg05724997 | cg23885005 | 0.999 | 0.998 | 0.999 | 1.50×10^-04^ | 3.38×10^-02^ | 0.994 | 0.991 | 0.998 | 1.15×10^-03^ | 0.971 | 0.948 | 0.995 | 1.89×10^-02^ |
| cg23913400 | cg24079672 | 0.886 | 0.846 | 0.927 | 1.81×10^-07^ | 4.21×10^-04^ | 0.827 | 0.760 | 0.899 | 7.68×10^-06^ | 0.720 | 0.529 | 0.981 | 3.72×10^-02^ |
| cg23913400 | cg24195535 | 0.886 | 0.833 | 0.943 | 1.34×10^-04^ | 3.17×10^-02^ | 0.883 | 0.829 | 0.940 | 1.08×10^-04^ | 0.782 | 0.642 | 0.953 | 1.50×10^-02^ |
| cg26869617 | cg27645750 | 0.934 | 0.901 | 0.968 | 1.57×10^-04^ | 3.44×10^-02^ | 0.932 | 0.890 | 0.976 | 2.60×10^-03^ | 0.888 | 0.803 | 0.983 | 2.22×10^-02^ |

# Table S7. Potential targeted drug for epigenetic predictors derived from DrugBank database.

| **Gene symbol** | **Drug name** | **Drug group** | **Change** | **Immunity-related Drug** |
| --- | --- | --- | --- | --- |
| *ATIC* | Methotrexate | Approved | downregulated | ✔ |
| *CCR2* | Alitretinoin | Approved Investigational | downregulated | ✔ |
|  | Cyclosporine | Approved Investigational Vet Approved | upregulated | ✔ |
|  | Decitabine | Approved Investigational | upregulated | ✔ |
|  | Methotrexate | Approved | downregulated | ✔ |
|  | Tretinoin | Approved Investigational Nutraceutical | upregulated | ✔ |
| *DLC1* | Arsenic trioxide | Approved Investigational | upregulated | ✔ |
|  | Cyclophosphamide | Approved Investigational | upregulated | ✔ |
|  | Cyclosporine | Approved Investigational Vet Approved | downregulated | ✔ |
|  | Formaldehyde | Approved Vet Approved | downregulated | ✔ |
|  | Irinotecan | Approved Investigational | downregulated | ✔ |
|  | Methotrexate | Approved | upregulated | ✔ |
|  | Panobinostat | Approved Investigational | upregulated | ✔ |
|  | Tretinoin | Approved Investigational Nutraceutical | upregulated | ✔ |
| *EIF2AK2* | Cyclosporine | Approved Investigational Vet Approved | upregulated | ✔ |
|  | Cisplatin | Approved | downregulated | ✔ |
|  | Formaldehyde | Approved Vet Approved | downregulated | ✔ |
| *FOXO1* | Arsenic trioxide | Approved Investigational | upregulated | ✔ |
|  | Belinostat | Approved Investigational | upregulated | ✔ |
|  | Cyclosporine | Approved Investigational Vet Approved | downregulated | ✔ |
|  | Dexamethasone | Approved Investigational Vet Approved | upregulated | ✔ |
|  | Entinostat | Investigational | upregulated | ✔ |
|  | Formaldehyde | Approved Vet Approved | downregulated | ✔ |
|  | Hydrocortisone | Approved Vet Approved | upregulated | ✔ |
|  | Panobinostat | Approved Investigational | upregulated | ✔ |
|  | Tretinoin | Approved Investigational Nutraceutical | upregulated | ✔ |
| *GAPDH* | Dactinomycin | Approved Investigational | upregulated | ✔ |
|  | Decitabine | Approved Investigational | upregulated | ✔ |
| *ITPR1* | Bexarotene | Approved Investigational | upregulated | ✔ |
|  | Dasatinib | Approved Investigational | upregulated | ✔ |
|  | Dexamethasone | Approved Investigational Vet Approved | upregulated | ✔ |
|  | Entinostat | Investigational | upregulated | ✔ |
|  | Formaldehyde | Approved Vet Approved | upregulated | ✔ |
|  | Methotrexate | Approved | upregulated | ✔ |
|  | Tamoxifen | Approved | upregulated | ✔ |
|  | Tretinoin | Approved Investigational Nutraceutical | downregulated | ✔ |
|  | Vincristine | Approved Investigational | upregulated | ✔ |
|  | Vorinostat | Approved Investigational | upregulated | ✔ |
| *NBR1* | Formaldehyde | Approved Vet Approved | upregulated | ✔ |
|  | Methotrexate | Approved | upregulated | ✔ |
| *NFE2L2* | Cyclosporine | Approved Investigational Vet Approved | upregulated | ✔ |
|  | Medroxyprogesterone acetate | Approved Investigational | upregulated | ✔ |
|  | Panobinostat | Approved Investigational | upregulated | ✔ |
|  | Tretinoin | Approved Investigational Nutraceutical | downregulated | ✔ |
|  | Tretinoin | Approved Investigational Nutraceutical | upregulated | ✔ |
| *PELP1* | Thimerosal | Approved | downregulated | ✔ |
| *PRKCQ* | Entinostat | Investigational | upregulated | ✔ |
| *SPNS1* | Formaldehyde | Approved Vet Approved | upregulated | ✔ |
| *TBK1* | Cyclosporine | Approved Investigational Vet Approved | upregulated | ✔ |
| *TBK1* | Formaldehyde | Approved Vet Approved | downregulated | ✔ |

**Figure S1.** Quality control processes for DNA methylation data.


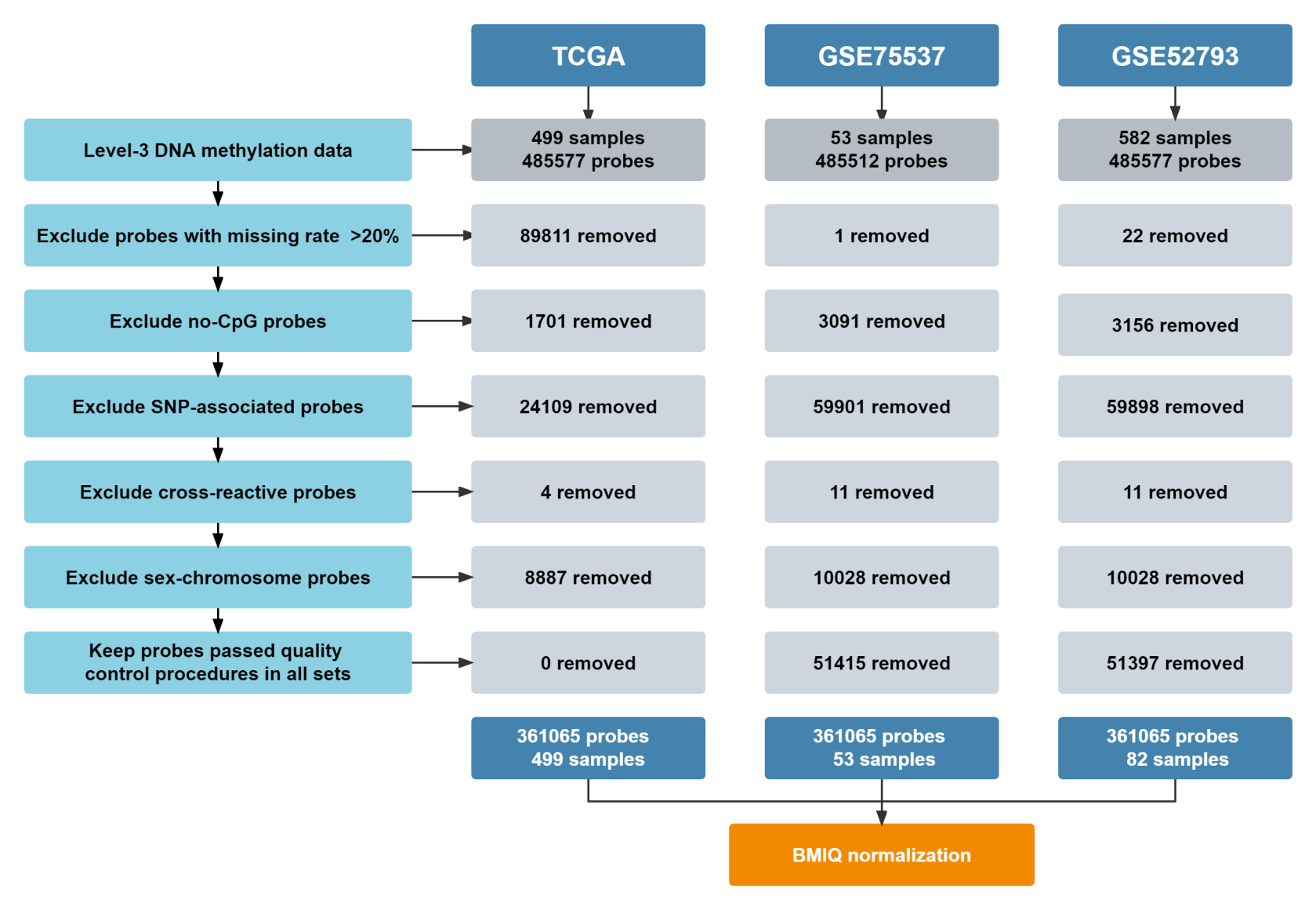


**Figure S2.** Subgroup analyses of ATHENA score stratified by site of resection or biopsy diagnoses. (A) Hazard ratio is used to evaluate the association between ATHENA score and HNSCC survival. The AUC is used to measure the prediction accuracy of ATHENA for (B) 36-month and (C) 60-month survival prediction.


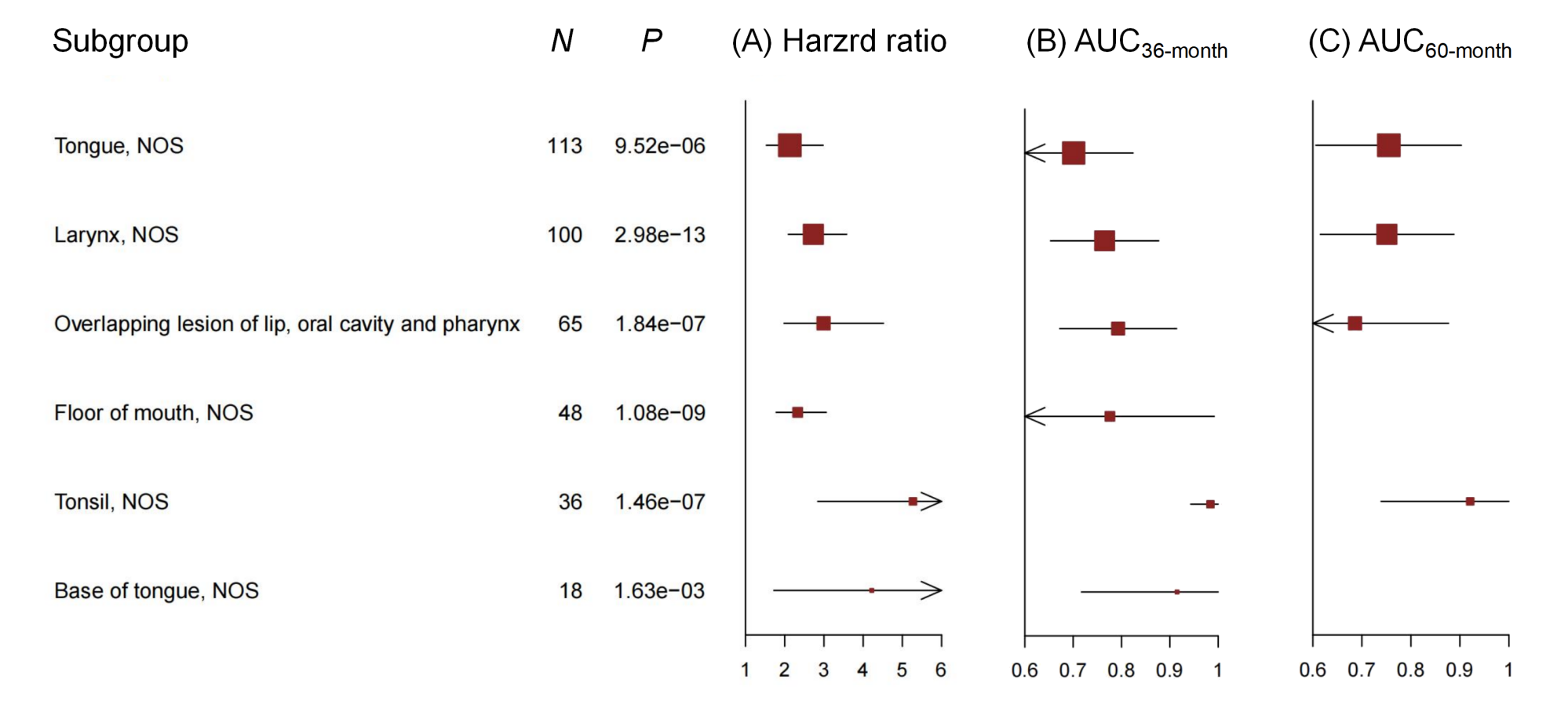


**Figure S3.** Prediction accuracy of ATHENA model in the internal testing set (GSE75537). (A) ROC curves of 36-month survival prediction and 60-month survival prediction. (B) The relative risk (*HR*) values of clinical variable and epigenetic score of ATHENA by multivariate Cox regression analysis. The horizontal lines in the color module represent the confidence interval of each factor.


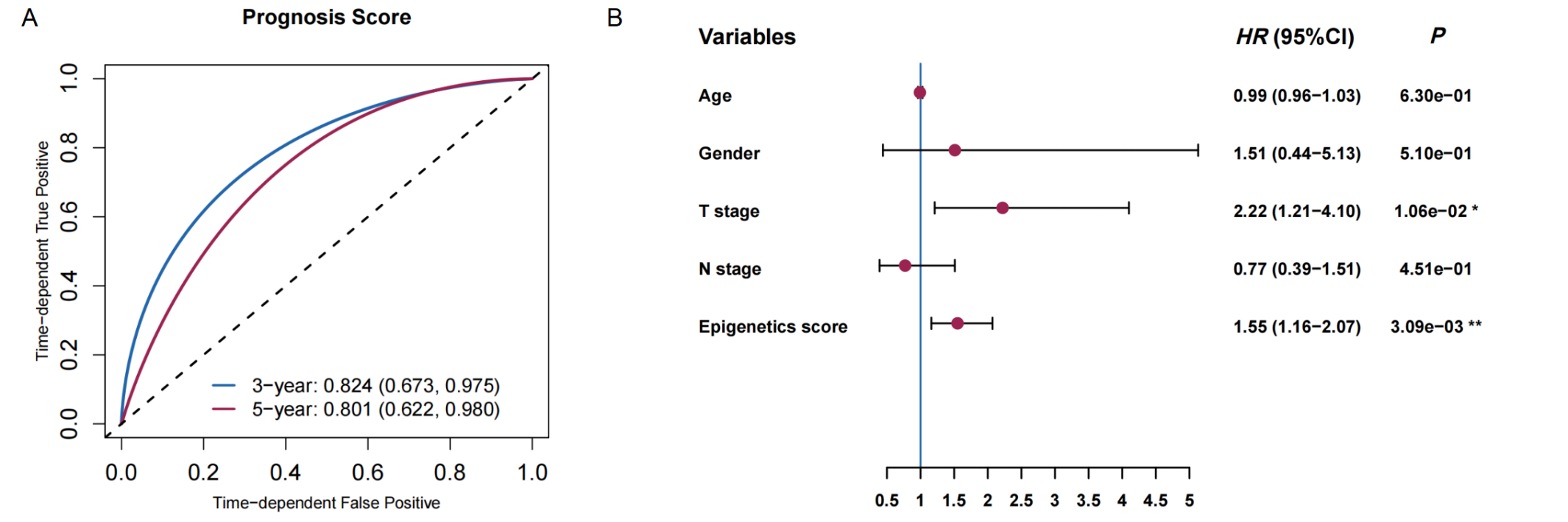


**Figure S4.** Prediction accuracy of ATHENA model in the external testing set (GSE52793). (A) ROC curves of 36-month survival prediction and 60-month survival prediction. (B) Kaplan-Meier survival curves for HNSCC patients with low and high epigenetic score of ATHENA. *P* values were derived from Harrington-Fleming test with parameters: (*p* = 0, *q* =1) and (*p* = 1, *q* =1). The former one was designed for late effects of variable during the follow-up and the later one was designed for both early and late effects of variable during the follow-up.


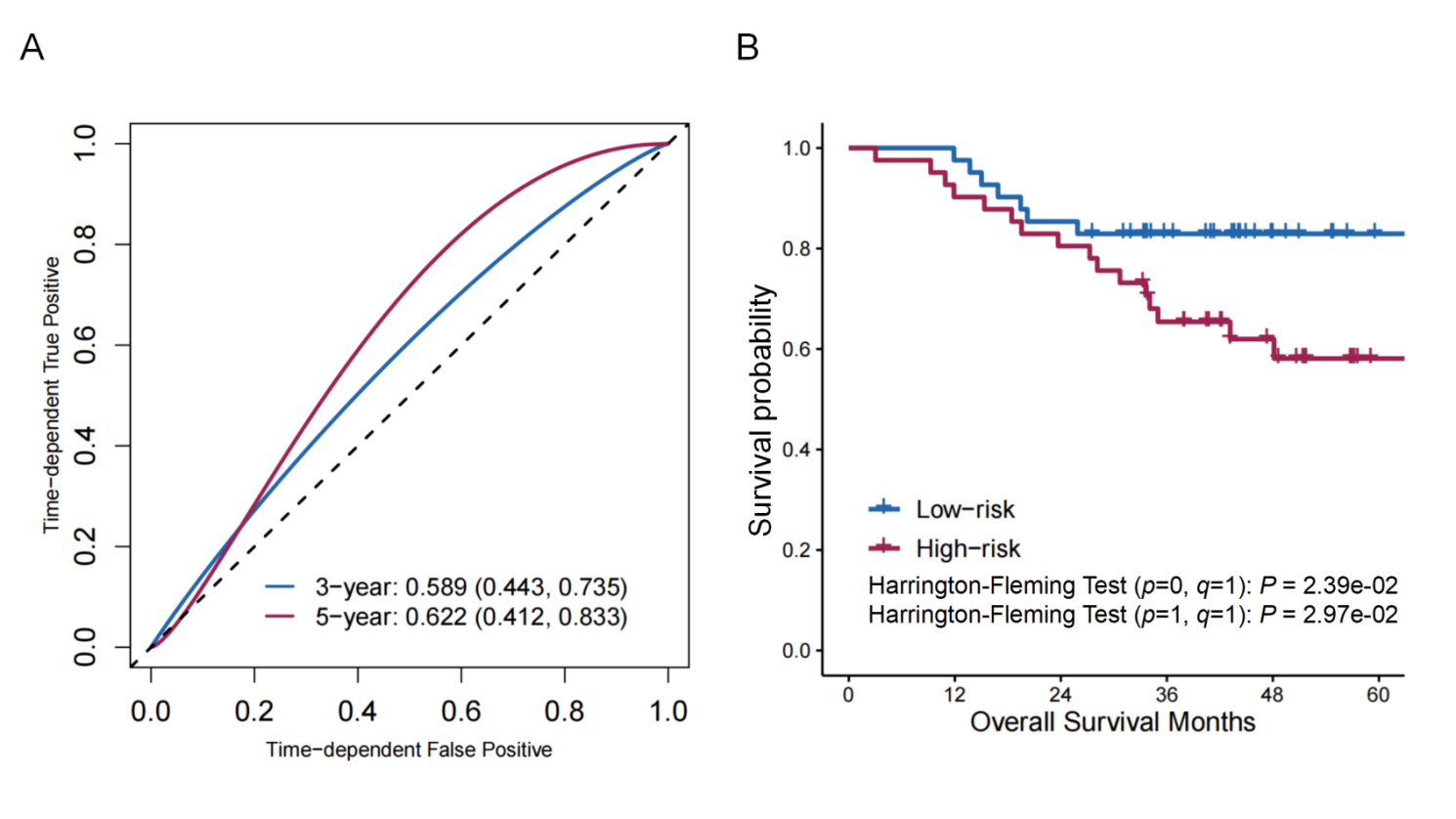


**Figure S5.** The correlation analysis between epigenetic score of ATHENA and pattern of tumor immune microenvironment (TIME).


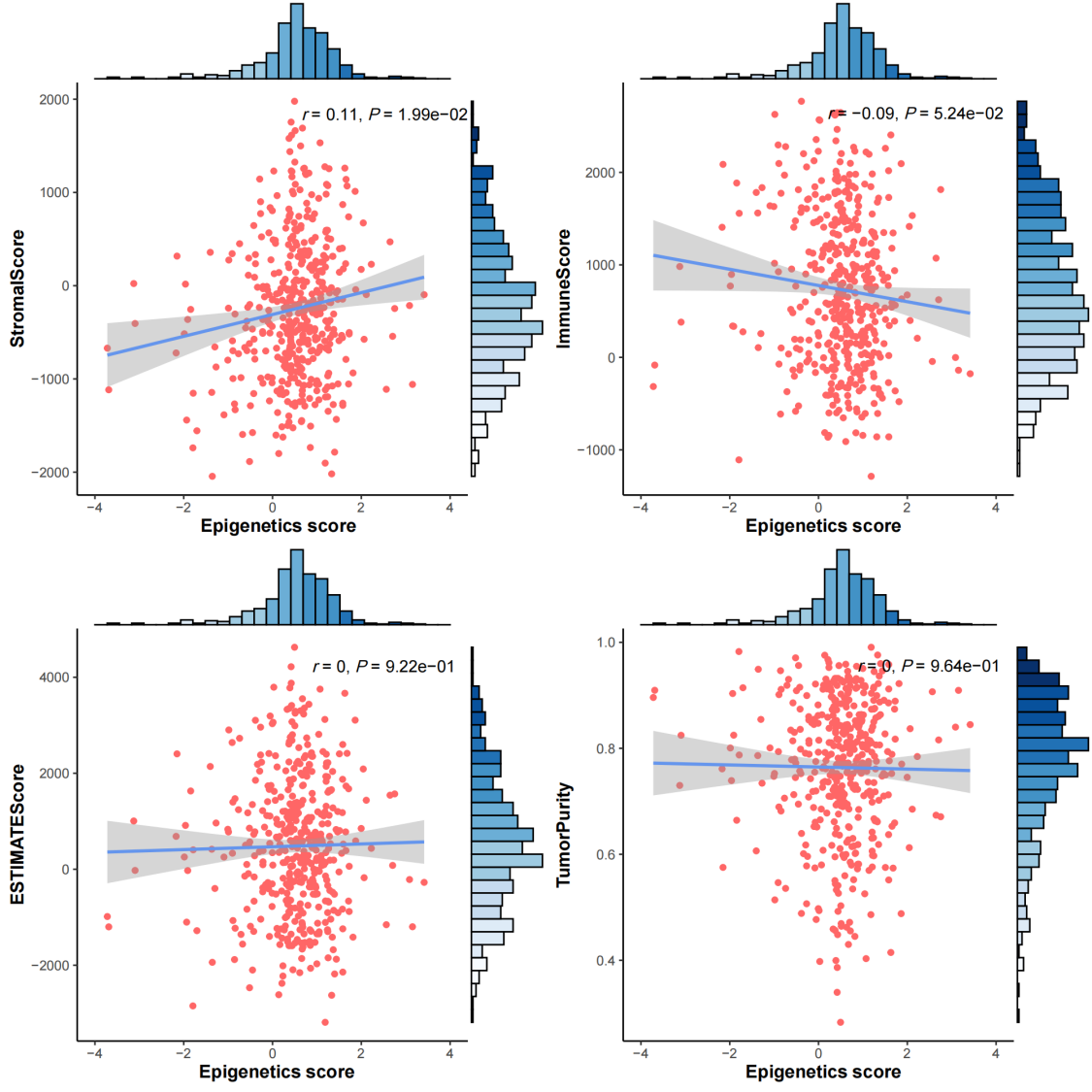

Supplement: Supplementary file 1 — Additional file 1. Supplementary File. [file 13148_2023_1501_MOESM1_ESM.docx]
